# Understanding COVID-19 nonlinear multi-scale dynamic spreading in Italy

**DOI:** 10.1007/s11071-020-05902-1

**Published:** 2020-09-01

**Authors:** Giuseppe Quaranta, Giovanni Formica, J. Tenreiro Machado, Walter Lacarbonara, Sami F. Masri

**Affiliations:** 1 Department of Structural and Geotechnical Engineering, Sapienza University of Rome, via Eudossiana 18, Rome, Italy; 2 Department of Architecture, University of Rome Tre, via Madonna dei Monti 40, Rome, Italy; 3 Department of Electrical Engineering, Institute of Engineering, Polytechnic of Port, Rua Dr. Antònio Bernardino de Almeida, 431, 4249-015 Porto, Portugal; 4 Department of Civil Engineering, University of Southern California, 3620 S. Vermont Ave, KAP 210, MC 2531, Los Angeles, CA 90089-2531 USA

**Keywords:** COVID-19, Compartmental model, Logistic regression, Nonlinear infection dynamics, Parametric identification, Computational intelligence

## Abstract

The outbreak of COVID-19 in Italy took place in Lombardia, a densely populated and highly industrialized northern region, and spread across the northern and central part of Italy according to quite different temporal and spatial patterns. In this work, a multi-scale territorial analysis of the pandemic is carried out using various models and data-driven approaches. Specifically, a logistic regression is employed to capture the evolution of the total positive cases in each region and throughout Italy, and an enhanced version of a SIR-type model is tuned to fit the different territorial epidemic dynamics via a differential evolution algorithm. Hierarchical clustering and multidimensional analysis are further exploited to reveal the similarities/dissimilarities of the remarkably different geographical epidemic developments. The combination of parametric identifications and multi-scale data-driven analyses paves the way toward a closer understanding of the nonlinear, spatially nonuniform epidemic spreading in Italy.

## Introduction

The coronavirus disease 2019 (COVID-19) is a highly infectious disease associated with SARS-CoV-2 virus leading to a Severe Acute Respiratory Syndrome which has affected 22,683,769 confirmed patients and caused 793,773 deaths worldwide as of August 21, 2020 [1].

After the officially reported outbreak in China in December 2019, COVID-19 has spread across the globe, at a faster rate than expected, to the level of a global pandemic causing health emergencies, huge economic losses, and social instabilities worldwide. Governments have been faced with new challenges such as quick enforcement of severe control measures (case isolation, social distancing, travel restrictions, and quarantine of local or national magnitude) to slow down the virus spreading and prevent a collapse of their healthcare systems, which would have caused a significantly larger number of deaths. These governmental decisions were generally supported by data-driven estimates put together by groups of expert virologists, epidemiologists, and health managers. At the heart of the matter, modeling approaches with predictive capability for infectious diseases such as COVID-19 are critical to make well-informed decisions which can have large-scale future effects.

There is a huge literature on mathematical modeling in epidemiology starting from the early work of [2], going through the compartmental models of [3–5] laying down the foundations of modern epidemiology, up to the current data-driven approaches (see, e.g., [6] addressing COVID-19).

Modeling approaches should be as accurate as possible in describing the disease spreading, over time and across different spatial scales, to meaningfully simulate the large-scale spreading and to design more effective and less costly control measures (e.g., local lockdowns of towns or counties or states instead of nation-wide lockdowns with severe economic losses). Most of the initial studies on COVID-19 [7–10] addressed aggregate data from Wuhan, China, where the virus apparently originated. According to [11], the very early infections were identified in Wuhan at the beginning of December 2019 and were controlled by the end of February 2020 following strong government lockdowns and restrictions. The fatality rate was estimated to be 3.8%, although the actual number is likely to be lower since the number of infected individuals was heavily underestimated, and no account was taken of the large number of asymptomatic cases. In these studies, among the essential epidemiological parameters estimated from data in Wuhan, $R_0$ (the basic reproduction number that indicates how contagious an infection disease is [12]) was found to be in the range of 2 to 3. Liu et al. [13] estimated $R_0=2.7$, larger than the earlier SARS epidemic reproduction numbers. On the other hand, Kucharski et al. [14] estimated that the lockdown and travel restrictions resulted in a decrease of $R_0$ from 2.35 to 1.05. Several works addressed the COVID-19 spreading in other areas of China and the world. A key study in the UK [15], based on the assumption $R_0=2.4$, suggested mitigation strategies for various countries, mainly USA and UK, with the objective of “flattening the curve” of cumulative infections. Notwithstanding the importance played by $R_0$ in policy making about measures designed to counteract epidemic spreading, the estimates of this parameter can be controversial given the large scattering exhibited by this parameter depending on how the estimates are performed and the setting of the initial assumptions. Impactful decisions depend on the estimates of $R_0$, in particular, relying on whether this is above or below the threshold value equal to 1, which discriminates between epidemic spreading and infection extinction. In Italy, some key studies [16] showed that the presence of a large class of asymptomatic infectives severely modifies the estimates of $R_0$ by as much as 5/2. According to [16], this underestimation could explain why most of the health systems were surprised by the rapid initial growth of the COVID-19 infections. Only later large-scale epidemiological studies recently conducted in Italy [17] showed the crucial role played by the substantial set of asymptomatic infectives.

In the context of compartmental models employed to describe the COVID-19 spreading, in [10] the population was divided into: susceptible subjects (*S*), had close contacts (*C*, those exposed to infected subjects/pathogen but not necessarily infected), latent (*E*, infected and infectious but asymptomatic), infected and symptomatic (*I*), recovered (*R*), and dead (*D*). The transmissibility of SARS-CoV-2 was modeled by two separate parameters, the social transmissibility factor $\beta$, which measures the probability of having close contact with infectious subjects, and the pathologic transmissibility, which measures the probability of an individual developing COVID-19 upon contact with the pathogen. The model was established based on demographic and COVID-19 epidemiological data in Wuhan. Data from Italy, the UK, and the USA were shown to fit well the model. A wealth of different compartmental models were recently proposed to understand the influence of asymptomatic individuals and the effects of control measures on the evolution of the disease [18], SEIR models combined with particle swarm optimization algorithm for parameter optimization [19, 20], a SAIR model in the context of social networks [21], a SEIRD model with classical and fractional-order derivatives based on data in Italy to show that the fractional-order model has less RMS error than the classical one [22].

In several studies on COVID-19, logistic regression (LR) methods were widely adopted for predictions, following a well-established stream of medical research for modeling the relationship between multiple independent variables and a categorical dependent variable (see, e.g., [23]). LR approaches have been tailored to identify several risk factors for COVID-19 pandemic diseases. To cite a few, Song et al. [24] performed multivariate LR analyses aiming to construct a diagnostic model that allows for the quick screening of highly suspected patients using easy-to-get variables. By employing backward stepwise selection and bootstrap resampling, Xie et al. [25] developed and validated a multivariable LR model that, on the basis of nine variables commonly measured in acute settings, predicts inpatient mortality in positive patients using data collected retrospectively from Tongji Hospital in Wuhan. With the aim of alleviating the limitations of medical resources, Bai et al. [26] combined traditional LR with deep learning-based methods, so as to devise a prediction model suitable for finding out the mild patients prone to become severe/critical cases and thus get timely treatments. On the same research line, Gong et al. [27] merged LR into a least absolute shrinkage and selection operator algorithm to construct a nomogram for risk prediction in the train cohort.

In other fully data-driven studies, Machado and Lopes [28] carried out statistical comparison and visualization of country-based COVID-19 data using data on the number of infected people over time. A comparison of the pandemic evolutions in different countries was performed using hierarchical clustering and multidimensional scaling which uncovered the emergence of patterns highlighting the main characteristics of the underlying complex dynamics.

In studies closely related to our work (see [29]), analysis of data from Italy showed that considering solely the confirmed case counts would be misleading since the trends are very much affected by the number of daily tests which, in turn, change from region to region. Moreover, the authors of this paper stated “reporting statistics on the national level does not say much about the dynamics of the disease, which are taking place at the regional level.” This statement describes well the motivation of our work. Indeed, differently from most of previous studies on COVID-19 addressing large-scale, aggregate data on the disease spreading, the focus of the present paper is on the investigation of the multi-scale spreading across Italy, taking into account regional and national data from the database of the Italian Ministry of Health. The 21 regions of Italy are the first-level constituent entities with well-defined powers in key sectors such as health, urban planning, etc. With the exception of Valle d’Aosta, a mountainous autonomous region in the northwestern part of Italy, each region is divided into a number of provinces (i.e., counties) embodying a number of towns and villages. Thus, in our study, the macroscale is the whole country, the mesoscales are represented by territories covered by regions and provinces, while the microscale is represented by towns. Due to difficulties with the collection of data from provinces and towns, we employ aggregate data for Italy and its severely affected regions, here treated as the smaller scale. We aim to describe the different dynamical properties of the pandemic across these regions and propose a method to quantify the different contributions of the regional epidemic spreading to the national epidemic dynamics. Our objective is motivated by the simple evidence that the COVID-19 spreading affects, in very different ways, different geographical regions, as well as people with different sex, age, health conditions with specific pathologies, and other characteristics. The multi-scale quantification is based on consideration of the different evolutions of the epidemiological variables rescaled with respect to the regional population or the national population. Again, in our approach, the global scale corresponds to the national scale, while the considered local dynamic scales represent the regional scales.

To provide a quick overview, the milestones of the COVID-19 pandemic in Italy are listed in Table 1 (data processed up to May 13, 2020) in terms of outbreak and policy enforcements.

**Table 1:** Milestones of the COVID-19 pandemic in Italy

Date	Event	Notes
Jan 30, 2020	First confirmed positive cases	Two Chinese tourists in Rome
Jan 30, 2020	International flights restriction	Flights to and from China closed
Jan 31, 2020	National emergency statement	
Feb 18, 2020	First secondary transmission case	Codogno (Lodi, Lombardia)
Feb 23, 2020	Municipal lockdown measures	Municipalities with outbreaks closed
March 8, 2020	Regional lockdown	Lombardia and 14 provinces closed
March 9, 2020	National lockdown	First national lockdown enforced
March 22, 2020	National lockdown	Stricter national lockdown enforced
May 4, 2020	Lockdown removal	Regional mobility allowed

A detailed study of the spreading across the main affected regions of Italy is carried out via parameter identification, using real data and their fitting with a SIR-type epidemiological model.

We selected one of the recently proposed SIR-type compartmental models to identify the epidemiological parameters of the multi-scale virus spreading in Italy. As known, the state variables represent the population numbers in various stages of the infectious disease progression. The classical SIR model (see, e.g., [3, 30, 31]) concerns averaged equations for a population of “equivalent” individuals in three states, namely susceptible (of infection), infected and infective, and removed (from the infective dynamics). Recently, Gaeta [32] proposed an enriched SIR-type model, referred to as A-SIR, which accounts also for the time evolution of the asymptomatic infectives, registered or unregistered recovered. Such a model was further revised by Paggi [33] to account for the evolution of deceased individuals connected to the number of current infected. This A-SIRD model proved to be quite accurate in describing the epidemic data in Italy.

We corroborate our findings, provided by the above-described method, with hierarchical clustering and multidimensional analysis unfolding the similarities/dissimilarities between the different regional scales in comparison with the national pandemic scale. This data-driven strategy involves various processes, starting with a first phase dealing with data validation, sorting, summarization and aggregation, and moving to a more insightful phase involving the dynamical analysis, report, and classification.

Clustering adopts a set of computational algorithms for grouping a set of items (also called objects) so that items in the same group (cluster) are more similar, according to a defined point of view, to each other, in contrast with items belonging to other groups. Here, hierarchical clustering (see, e.g., [34–36]) and multidimensional scaling techniques (see, e.g., [37–40]) are employed. The first seeks to construct a hierarchy of clusters. The second is a computational tool for visualizing the similarity between items and translating the information into a configuration of points mapped into an abstract Cartesian space. The core of the adopted schemes is the choice of the distance measure (see, e.g., [41]) and the implementation of some optimization indices for constructing graphical representations in terms of dendrograms and trees and multidimensional scaling (MDS) plots.

## Analysis of COVID-19 pandemic dynamics in Italy

### Data collection

**Fig. 1:**
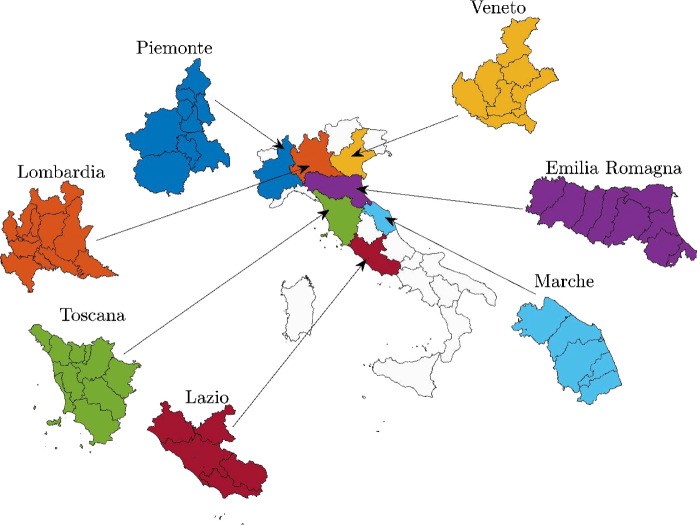
Map of Italy highlighting in different colors the analyzed regions and their provincial boundaries

Geographical maps employed in the present study (including administrative boundaries at provincial and regional level) are retrieved from the official Web site of the National Institute of Statistics. The maps (WGS 84, UTM zone 32N, 2020 edition) are public and available in shapefile format (last access on May 13, 2020). Figure 1 shows the map of Italy and its regions. The analyzed regions (Lombardia, Piemonte, Veneto, Liguria, Emilia-Romagna, Toscana, Lazio) are highlighted by different colors which will be used consistently across all subsequent graphical representations.

Demographic data are also retrieved from the same Web site (last access on May 13, 2020). Most updated information at regional levels dates back to 2019. At provincial levels, updated data about the population are not available directly for all statistics, since the most recent sets date back to the 2011 national census.

Italian epidemic data adopted in the present study are provided by the Italian Department of Civil Protection through a public repository hosted on GitHub (last access on May 13, 2020). Besides information at the national level, epidemic data at both regional and provincial levels are also available. Specifically, at national and regional levels, the following sets of epidemic data have been updated daily since February 24, 2020: hospitalized patients with symptoms; intensive care patients; total hospitalized patients; home confinements; daily positive cases (i.e., sum of hospitalized patients and home confinements) denoted by *I*; new daily positive cases (i.e., difference between current total positive cases and the corresponding value of the previous day); recovered cases (sometimes referred to as cumulative cases) denoted by *R*; deceased cases indicated by *D*; total confirmed positive cases (sometimes referred to as cumulative cases) denoted by *N*; tests carried out; and tested people.

**Fig. 2:**
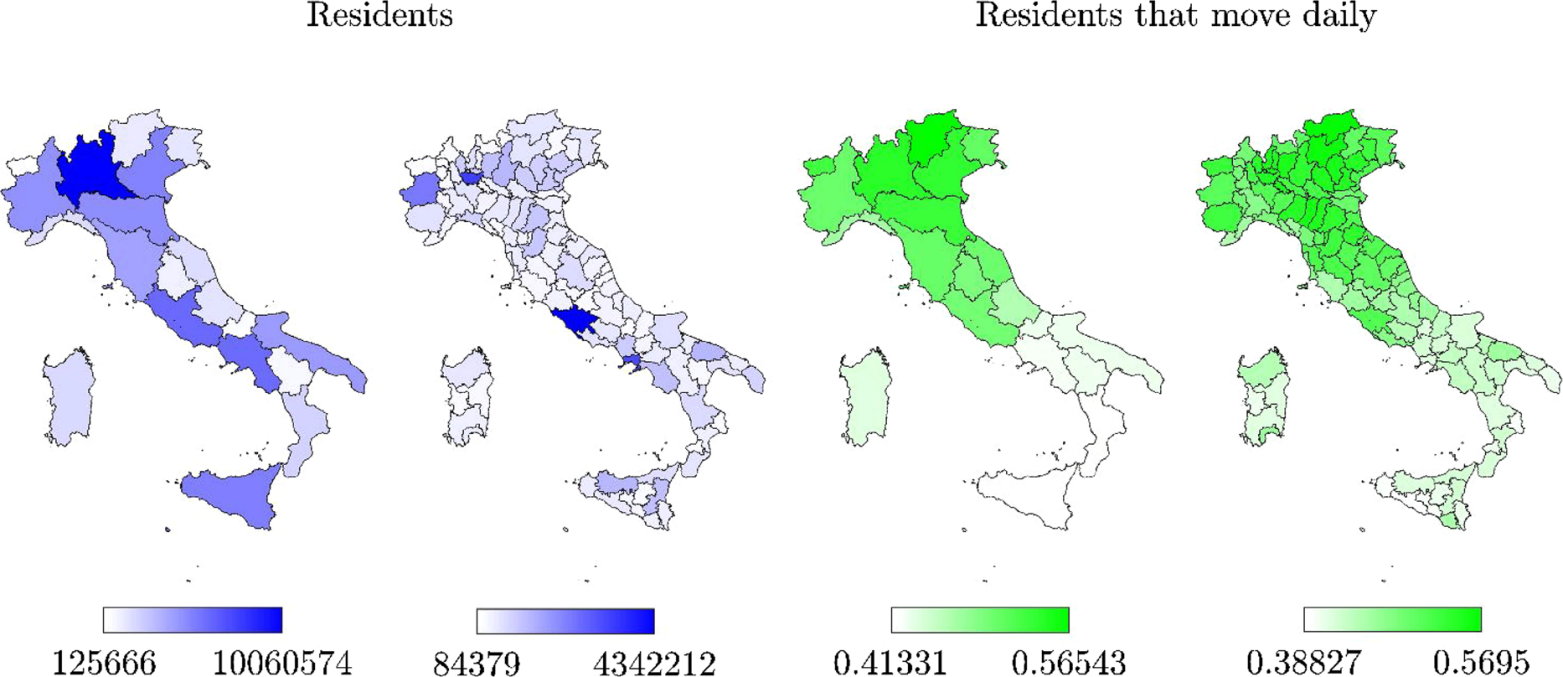
Italian regional and provincial maps of the population (residents $P_R$) and number of residents that move daily normalized with respect to the population

At the provincial level, only the total positive cases *N* have been daily updated since February 24, 2020. However, some data about the total positive cases provided by the Italian Department of Civil Protection are not assigned to a specific province. While the assignment of a positive case to a precise region is usually not affected by appreciable uncertainty, this turns out to be a more complicated or, in some cases, impossible task at the provincial level. In the present study, unassigned provincial data are not taken into account for data processing. This, in turn, is reflected into some differences that can be observed when elaborating the number of total positive cases at the regional and provincial levels. It is also to be noted that, at the regional level, data provided by the Italian Department of Civil Protection about the tested people are available since April 19, 2020.

### Data analysis

Before starting the COVID-19 pandemic analysis in Italy, meaningful demographic data are collected to help guide the interpretation of the multi-scale virus spreading. In this context, the number of residents at the regional and provincial levels, according to 2019 official data, is provided in Fig. 2. Moreover, Table 2 shows the number of residents for those regions deserving special consideration in the present study. The number of residents that move daily normalized with respect to the regional population is also provided in Fig. 2. The daily mobility is very important since it is clearly associated with a number of key parameters affecting the disease spreading such as the contact rate or social transmissibility factor. Moreover, the role played by air pollution in facilitating pandemic spreading and increasing the fatality rate has been largely investigated. Although the majority of existing studies agrees on the importance of this correlation, this is still a debated topic undergoing further investigations [42]. Although most updated mobility data at the regional level date back to 2019, data at the provincial level are not directly available for the same year. Therefore, the maps about the number of residents that move daily in Fig. 2 are elaborated using 2011 data, the most recent year in which this information is available at both the regional and provincial levels. According to 2019 data, the number of residents in Italy was 60,359,546. The region with the largest number of residents is Lombardia, where about 16.7% of the national population resides. Mobility data highlight the presence of two different areas in the country, i.e. near 60% of the residents move daily in the northern and central part of Italy, whereas this rate goes down below 40% in southern regions of Italy. There are other important factors which may help explain the different multi-scale spreading such as the pollution severity in different regions.

**Table 2:** Number of residents for some Italian regions and the whole country (2019 data)

Region	Number of residents $P_R$
Piemonte	4,356,406
Lombardia	10,060,574
Veneto	4,905,854
Emilia-Romagna	4,459,477
Toscana	3,729,641
Marche	1,525,271
Lazio	5,879,082
*Italy*	60,359,546

The processed data span the most critical phase of COVID-19 pandemic in Italy, i.e., the time interval March–April 2020. Particularly, for an improved visualization, the following maps refer to the time window between March 7, 2020 (i.e., a few days before the first lockdown started), and April 28, 2020 (i.e., a few days before lockdown measures started to be relaxed).

**Fig. 3:**
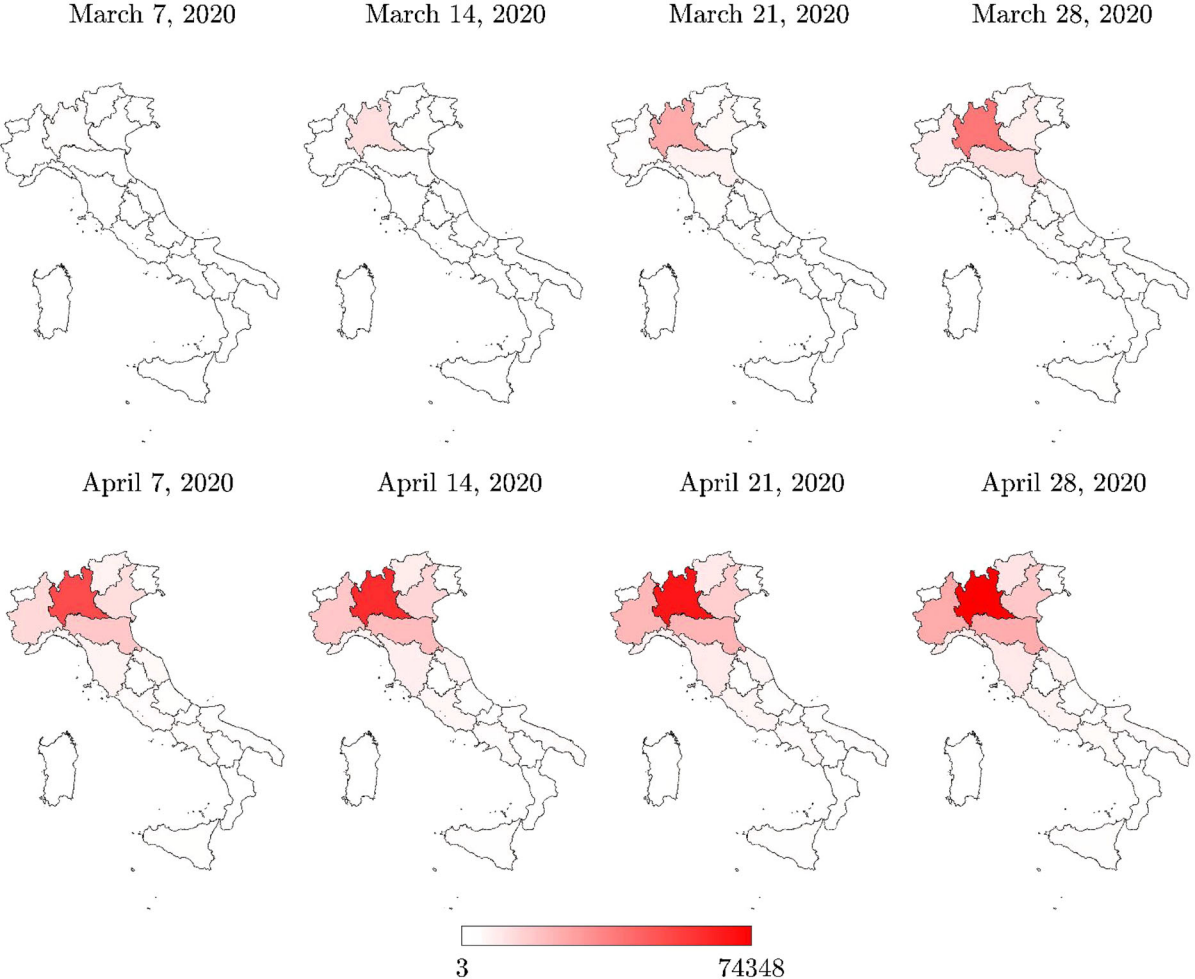
Italian regional map with the total positive cases $N_R$

**Fig. 4:**
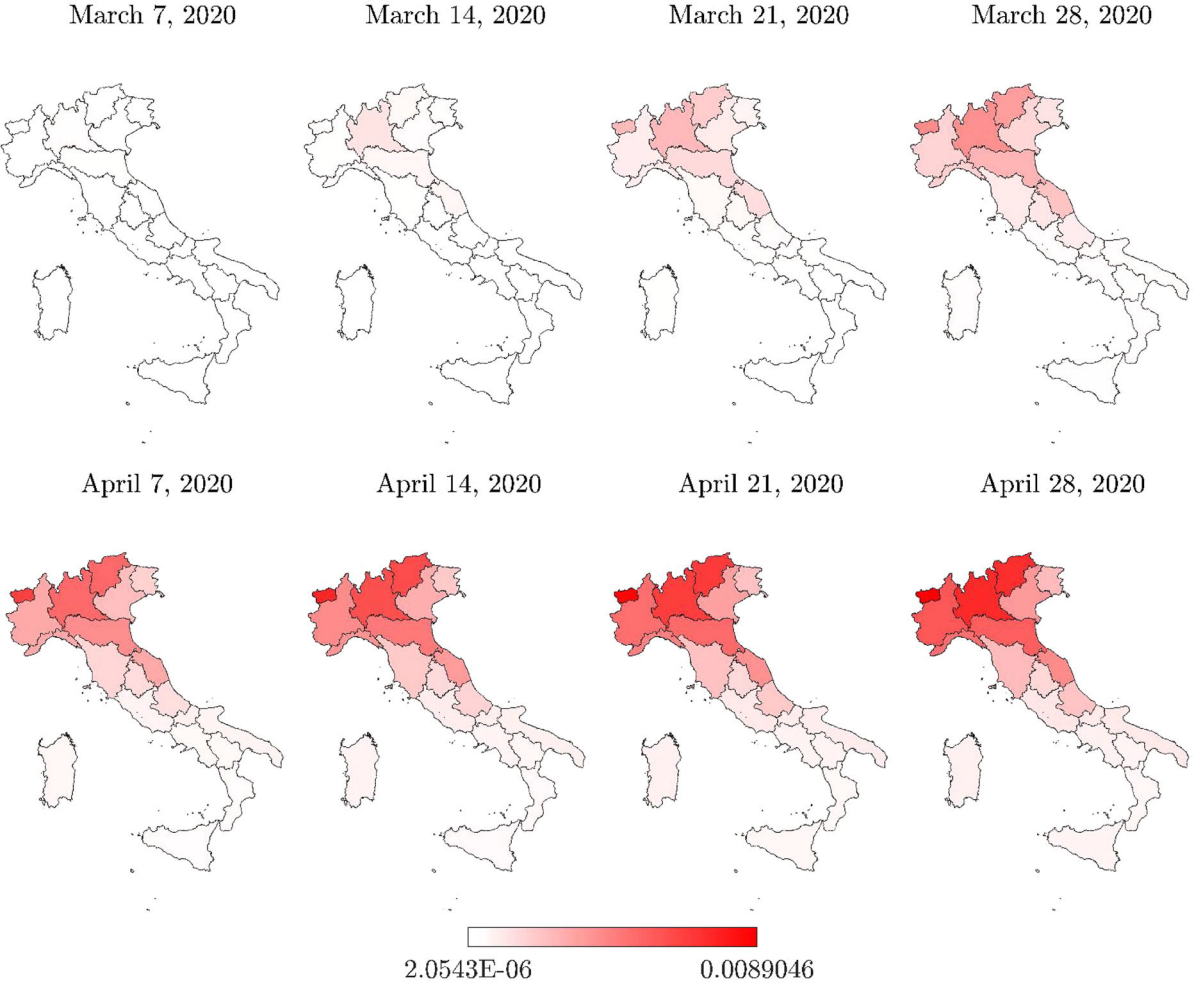
Italian regional map with the total positive cases normalized with respect to the number of residents $N_R/P_R$

**Fig. 5:**
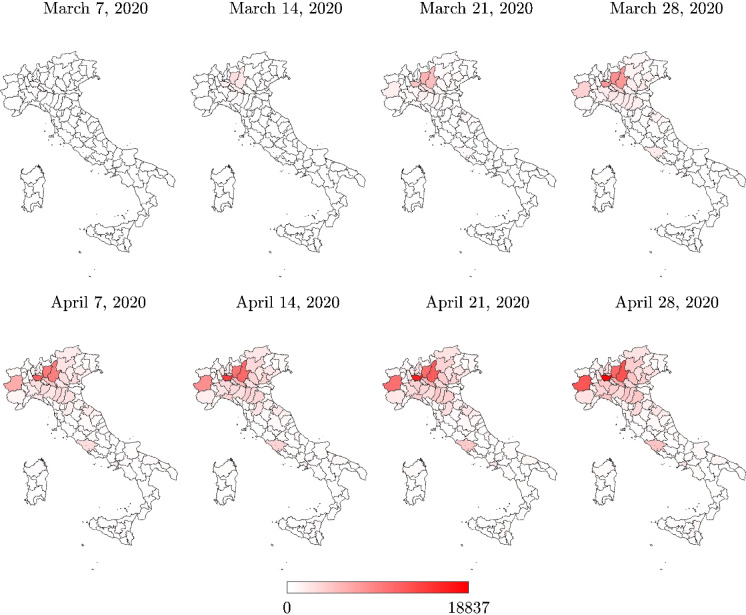
Italian provincial map with the total positive cases

**Fig. 6:**
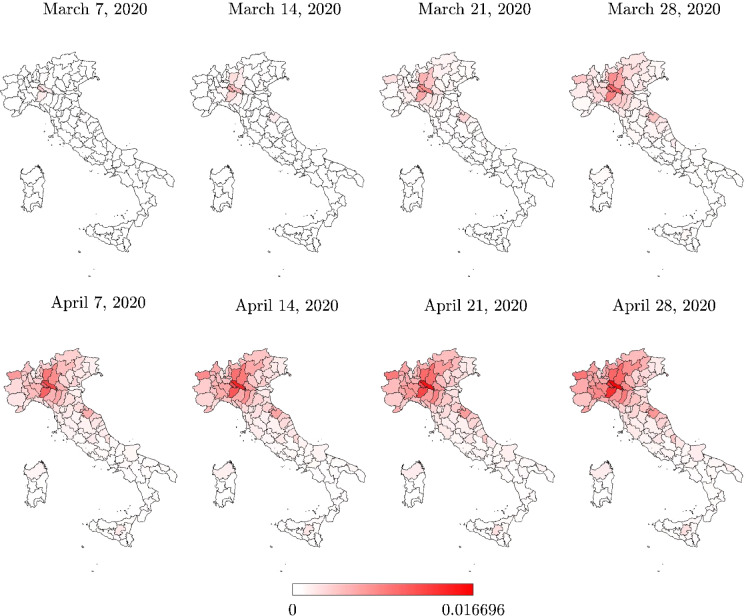
Italian provincial map with the total positive cases normalized with respect to the number of residents

Italian regional maps of the total positive cases are shown in Figs. 3 and 4. The total positive cases at the provincial level are presented in Figs. 5 and 6. Data about the total positive cases for some regions and the whole country are also listed in Table 3.

In the vicinity of the lockdown suppression of April 28, 2020, the maximum number of total positive cases at the regional level was achieved in Lombardia, but a high number of positive cases were also recorded in Piemonte, Veneto, and Emilia-Romagna, as can be seen in Table 3. In terms of total positive cases, these regions in northern Italy were the most severely affected by COVID-19, whereas some regions of the central part of Italy, such as Toscana, Marche, and Lazio, were marginally affected. Compared to these northern and central regions, COVID-19 spreading in southern Italy turned out to be far less significant.

Analysis of the total positive cases at the regional level, normalized with respect to the number of residents, provides another point of view about the COVID-19 pandemic dynamics. In fact, on the same date (April 28, 2020), the maximum value of the total positive cases per number of residents was achieved in Valle d’Aosta. Herein, it turns out that the number of total cases compared with the number of residents was equal to $8.90\permille$, thus slightly larger than the value recorded in Lombardia, and significantly higher than the corresponding values recorded in some other Italian regions that were identified as the most critical. In some regions, the number of total positive cases, normalized with respect to the number of residents, suggests a diffusion of the infection larger than what the raw number indicates, as it can be observed by comparing Figs. 3 and 4. For instance, the total number of positive cases recorded in Liguria, Trentino-Alto Adige, and Marche is rather low as compared to the regions that were identified as the most critical in the country (equal to 7,772, 6,523 and 6,175 total positive cases on April 28, 2020, respectively), but the corresponding value normalized with respect to the residential population can be similar or higher (equal to $5.01\permille$, $6.08\permille$ and $4.05\permille$ on April 28, 2020, respectively).

Three provinces in Lombardia experienced the highest number of total positive cases, namely Milano, Brescia, and Bergamo. Herein, the number of total positive cases recorded in the vicinity of the lockdown removal (April 28, 2020) was found to be equal to 18,837, 12,691, and 11,196, respectively. The province of Torino also experienced a large number of total positive cases on the same date, namely 12,564. Hence, the percent numbers of total positive cases (with respect to the residential population) in the provinces of Milano, Brescia, Bergamo, and Torino are $5.79\permille$, $10.02\permille$, $10.04\permille$  and $5.56\permille$  respectively (April 28, 2020). Although these provinces have been a concern for a long time due to the large number of total positive cases, the number of total positive cases, normalized with respect to the number of residents, once again provides a different point of view about the geographical areas which suffered the infection dynamics more severely, as it can be observed by comparing Figs. 5 and 6. In fact, the available data indicate that the province of Cremona in Lombardia experienced the largest number of total positive cases with respect to the size of the residential population, since 5993 total positive cases corresponding to $16.69\permille$ of the number of residents were recorded a few days before the lockdown removal (April 28, 2020). The total positive cases normalized with respect to the number of residents are also very high for the province of Piacenza in Emilia Romagna (3918 positive cases on April 28, 2020, corresponding to $13.64\permille$ of the number of residents) and the province of Lodi in Lombardia (2947 positive cases on April 28, 2020, corresponding to $12.80\permille$ of the number of residents). Generally, it can be noted that the total positive cases at provincial level, normalized with the number of residents, can exceed twice the value at the corresponding regional level. Moreover, the value at the regional level can turn out to be about 2.5 times larger than the national value.

**Fig. 7:**
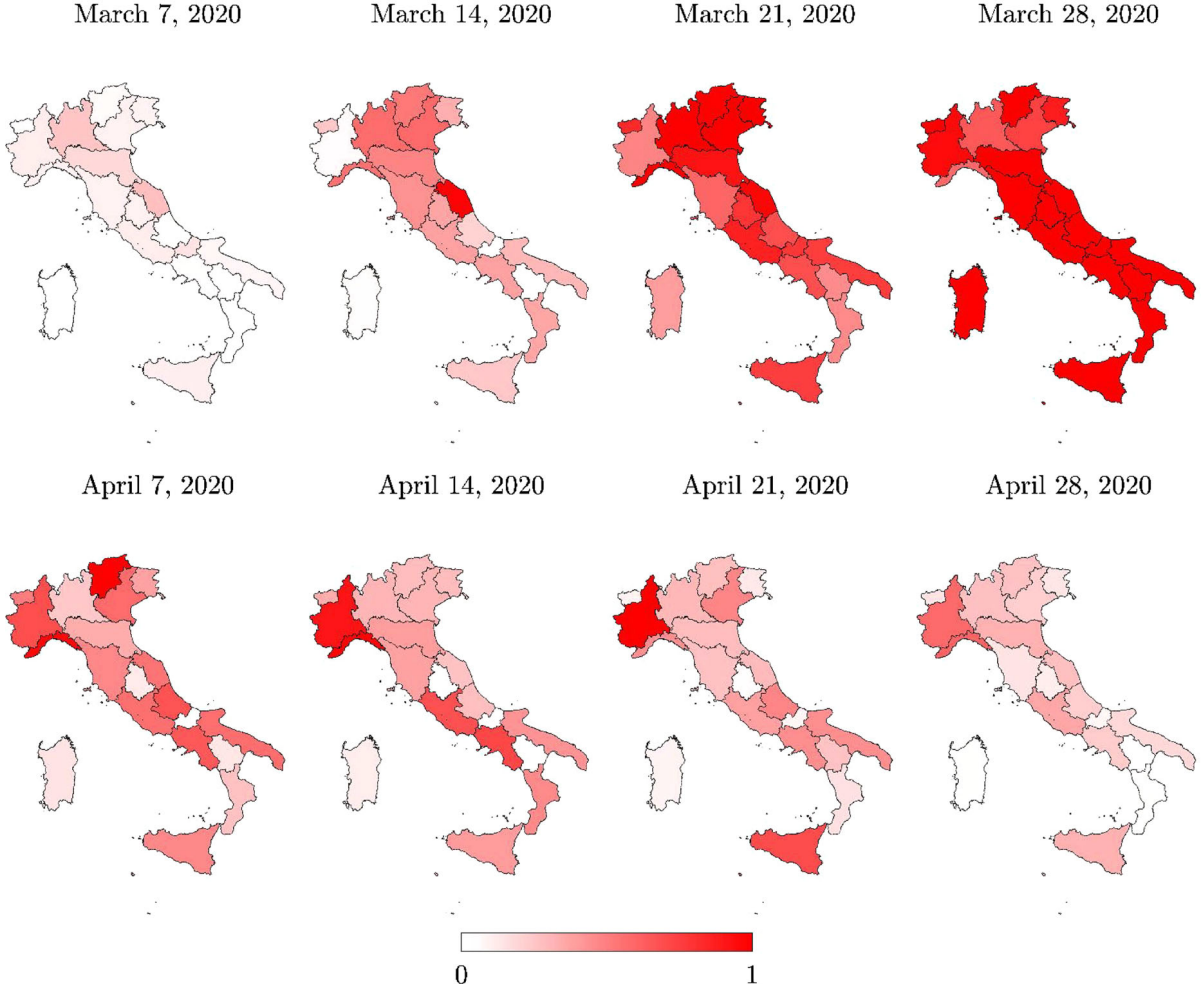
Italian regional map with the daily new positive cases normalized with respect to the corresponding peak value (limited to the selected set of dates)

**Fig. 8:**
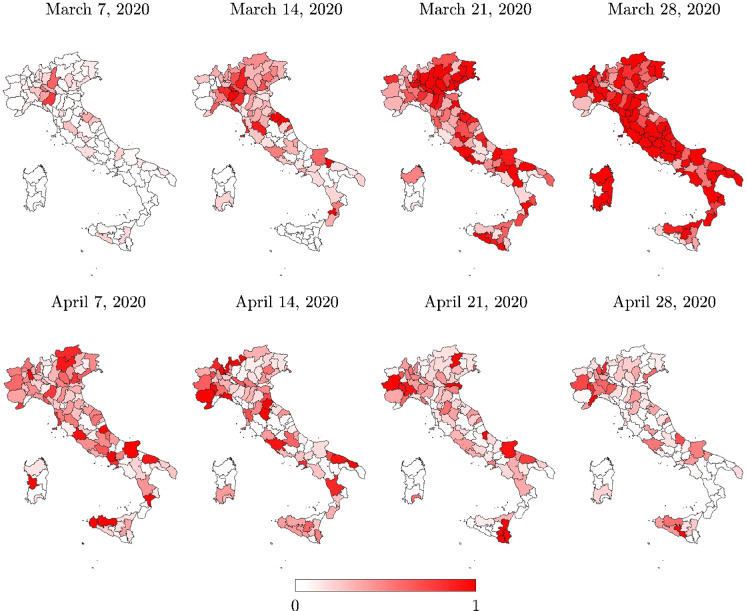
Italian provincial map with the daily new positive cases normalized with respect to the corresponding peak value (limited to the selected set of dates)

The analysis of the daily new positive cases also provides further insights into the infection transmission in time and space. To this end, regional information about the daily new positive cases is given in Fig. 7. At the provincial level, information about the daily new positive cases is shown in Fig. 8, herein computed for each province as the difference between current total positive cases and the corresponding value of the previous day, in agreement with the definition employed at the regional level. Note that the number of daily new positive cases at the provincial level is not directly communicated by the Italian Department of Civil Protection.

**Fig. 9:**
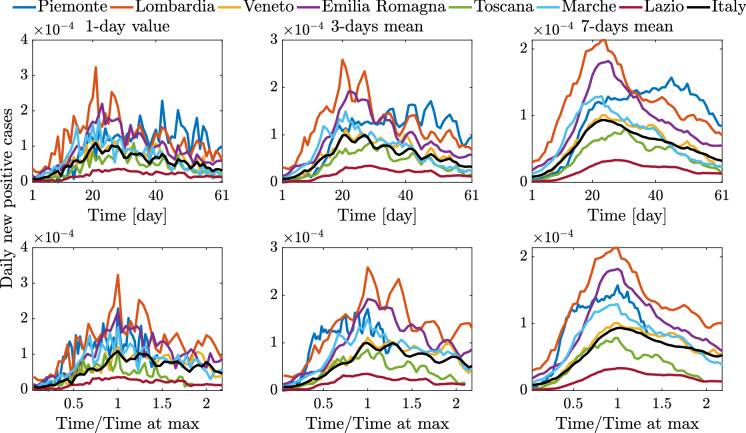
Time histories (single value and centering moving mean) of daily new positive cases scaled by the resident population for the selected Italian regions and Italy, between March 1, 2020 (day 1), and April 30, 2020 (day 61)

**Fig. 10:**
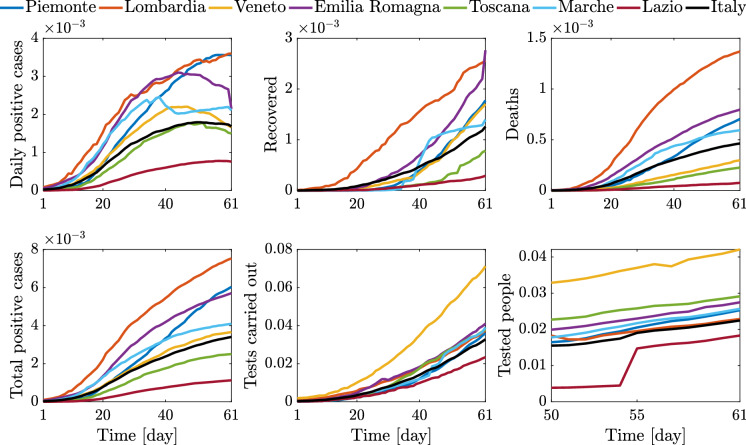
Time histories about epidemic and testing policy scaled by the resident population for the selected Italian regions and Italy, between March 1, 2020 (day 1), and April 30, 2020 (day 61)

Finally, time histories of the relevant indices for the selected regions and the whole country (starting from the first available recorded data until April 30, 2020) are also illustrated in Figs. 9 and 10.

Figures 7 and 8 show that almost all regions and provinces attained their peak of daily new positive cases within a few days after the most severe national lockdown measures were adopted, with the exception of a few regions, namely Marche and Piemonte. In particular, daily new positive cases close to the peak value started to be recorded in Marche a few days before the other regions in Italy. Conversely, daily new positive cases close to the peak value were recorded in Piemonte for quite some time beyond what was observed in the rest of the country. These outcomes are in general good agreement with the time histories of the daily new positive cases in Fig. 9.

**Table 3:** Total positive cases for each region ($N_R$ ) and for Italy (*N*), and their percentages with respect to both regional residents ($P_R$), and total cases *N*

Region	March 7, 2020			April 28, 2020		
	$N_R$	$N_R/P_R$ $\permille$	$N_R/N$%	$N_R$	$N_R/P_R$ $\permille$	$N_R/N$%
Piemonte	207	0.05	3.52	25,450	5.84	12.63
Lombardia	3420	0.34	58.13	74,348	7.39	36.90
Veneto	543	0.11	9.23	17,708	3.61	8.79
Emilia-Romagna	1010	0.25	17.17	24,914	5.59	12.36
Toscana	113	0.03	1.92	9231	2.47	4.58
Marche	207	0.14	3.52	6175	4.05	3.06
Lazio	76	0.01	1.29	6467	1.10	3.21
*Italy*	5883	0.10	100.00	201,505	3.34	100.00

Besides the trend of the epidemic data, Fig. 10 provides an interesting and useful overview about the implemented testing policy. Although the available time histories of tested people in Fig. 10 span a rather short period, they suggest that, with the sole exception of Veneto, by and large the considered regions have adopted a similar testing policy. That is, most regions carried out tests for a comparable percentage of residents, and such ratio has grown almost linearly in time at a constant rate. This, in turn, implies that significant differences in the number of positive cases among most regions are not significantly affected by the number of tested people, but they are an inherent feature of the pandemic dynamics at territorial level.

## Simulation of COVID-19 pandemic dynamics in Italy

We start delving into the epidemic data analysis employing a popular logistic regression model. This is done to see whether the different regional epidemic evolutions follow the usual pattern, whereby the initial stage of exponential growth is followed by a saturation stage.

### Logistic regression

Examples of logistic models employed in medicine are typically targeted to predict the presence or absence of a disease in relation to a variety of factors [43], a potential improvement that can be achieved after an intervention [44], whether newly explored variables can improve the predictive validity of already established models (e.g., [45]), but also to develop novel *r* statistical methods on the basis of ranked data [46].

Here, by recalling that *N* denotes the number of total positive cases, its time evolution obeys the following equation:

1 $$\begin{aligned} N(t) = \frac{K\,N_0\,e^{rt}}{K+N_0(e^{rt}-1)}\,, \end{aligned}$$

where *t* stands for time, $N_0$ is the initial value, *K* is the carrying capacity, and *r* represents the rate of increase in *N*.

Recall that the problem of employing confirmed cases to fit models is highly uncertain and is furthermore complicated by the fact that the fraction of cases that are confirmed is spatially heterogeneous and nonlinearly time-varying [47, 48].

**Fig. 11:**
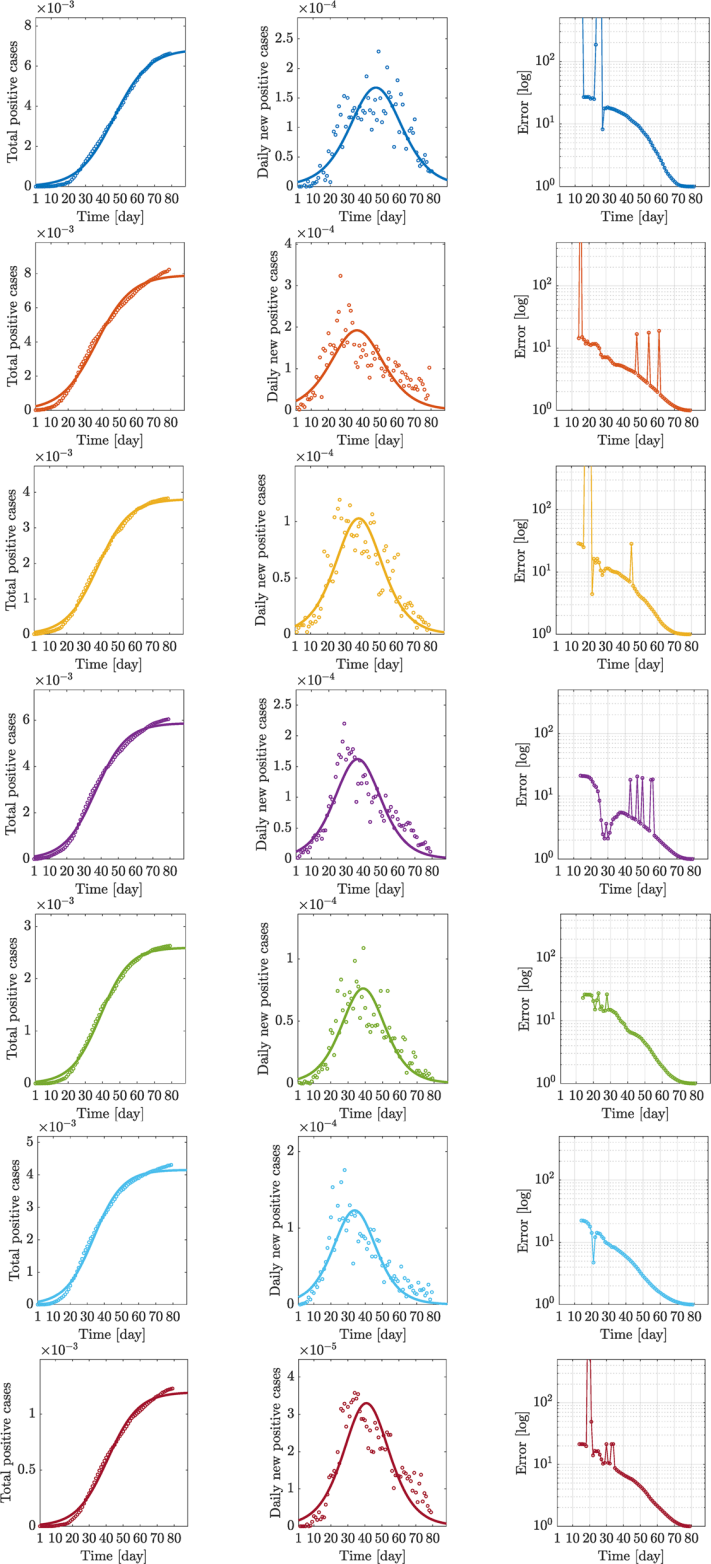
Logistic regression curves for Piemonte, Lombardia, Veneto, Emilia-Romagna, Toscana, Marche, Lazio: total positive cases, daily positive cases, and error evolution *e* (positive cases are normalized with respect to the number of residents)

The results for the targeted regions are reported in Fig. 11. The plots in the first column portray the comparison between real positive cases and its logistic curve *N*(*t*), while the plots in the second column compare real daily new positive cases with the analytic daily rate of *N*(*t*), i.e., ${\dot{N}}(t)$; finally, an error measure is introduced in the third column, aiming at measuring the accuracy of logistic curves for predicting the epidemic phenomena beyond the observed data.

**Fig. 12:**
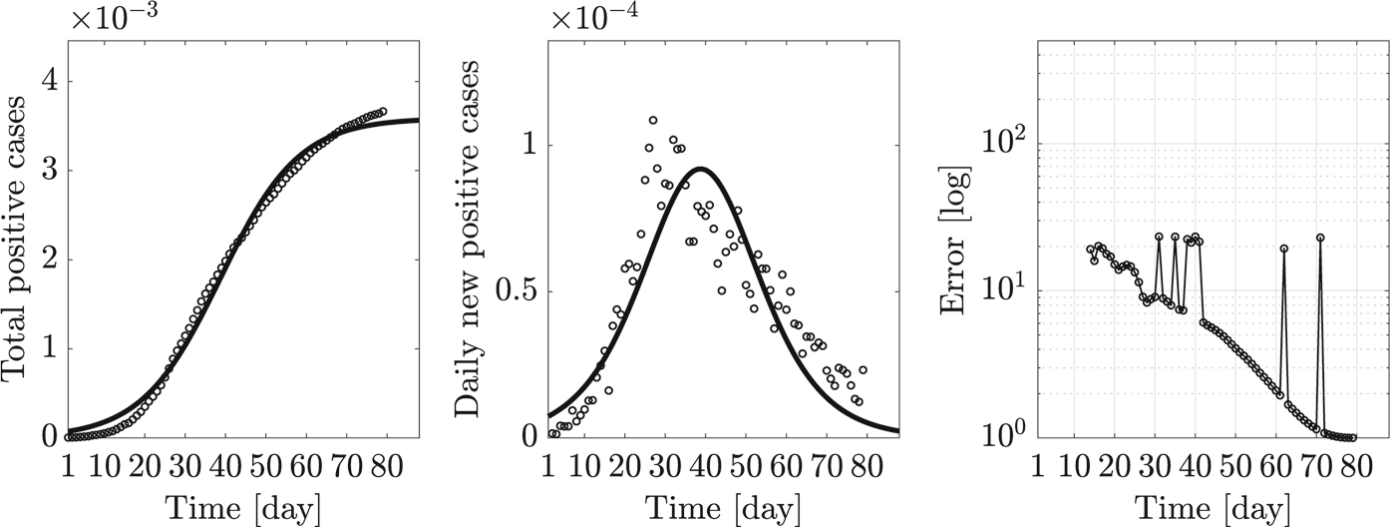
Logistic regression curves for Italy: total positive cases, daily positive cases, and error evolution (positive cases are normalized with respect to the number of residents)

In particular, the indicated error is the nondimensional mean squared error (MSE), varying in time as a discrete function, and obtained in the following way: starting from an initial day $k_0$, we measure the MSE error of a fitted curve with data available at the *k*th day, with $k=k_0+1,\,\ldots ,\,L$, and *L* the end day of analysis; such a daily error is then divided by the MSE value of the fitted logistic curve obtained over the complete sample of data. More specifically, denoting with $\varvec{\theta }_k=\{N_0,\,K,\,r\}_k$ the set of logistic-model parameter estimated from the data available at the *k*th day, with $N_k$ the corresponding fitted logistic function with $\varvec{\theta }_k$, the MSE error at the *k*th day, denoted by $m_k$, is computed as

2 $$\begin{aligned} m_k = {\left( \dfrac{1}{L}\sum _{j=1}^L{\left( N_k(t_j)-O(j)\right) ^2}\right) }^{1/2}\,, \end{aligned}$$

with *O*(*j*) denoting the value of observed data, i.e., the real number of confirmed cases for $j=1,\,\ldots ,\,L$. Its nondimensional value *e*(*k*), then reported in the third column of Fig. 11, is expressed as

3 $$\begin{aligned} e(k) = m_k/m_L. \end{aligned}$$

Note that *e*(*k*) tends to 1 as $k\rightarrow L$, i.e., at the end day *L* of the sample.

In all considered regions, such an error tends slowly to one, demonstrating a low capability of the logistic model to predict the actual epidemic evolution. Particularly in Lombardia (Fig. 11, second row, third column) and Emilia-Romagna (Fig. 11, fourth row, third column), and less markedly in Veneto (Fig. 11, third row, third column) and Lazio (Fig. 11, seventh row, third column), its evolution is noticeably not smooth, even far from the initial days. Moreover, all error curves exhibit changing trends around the lockdown days, as seen in the error oscillations approximately between the 15th and the 20th days.

On the other hand, the plots in the second column reporting the comparison between daily cases with the time derivative of *N*, ${\dot{N}}(t)$, allow to draw some conclusions about the dynamics represented by the logistic model. In fact, as shown in Fig. 11, while the logistic regression *N*(*t*) computed over the total positive cases definitely describes well their evolution, it poorly predicts the daily rate of the same data and then implicitly suggests the need of a dynamic model to better represent the infectious disease evolution.

Finally, the same computations carried out for each region are evaluated for the Italian national data and reported in Fig. 12.

### Nonlinear dynamic infection modeling

#### Compartmental model

The multi-scale COVID-19 pandemic in Italy is investigated employing a compartmental model chosen among the huge number of available formulations. Models with a high level of complexity are discarded, as they usually rely on a large number of parameters, which may hinder the real capability of such mathematical representations of describing the actual infection nonlinear dynamics. Moreover, all data required for the identification of a large set of model parameters might not be available, or their reliability may be largely questionable. On the other hand, the adopted nonlinear model should be able to reflect the principal aspects governing the overall infection dynamics. In this light, a wealth of studies have highlighted that asymptomatic infectives have a large influence on the COVID-19 pandemic dynamics.

**Fig. 13:**
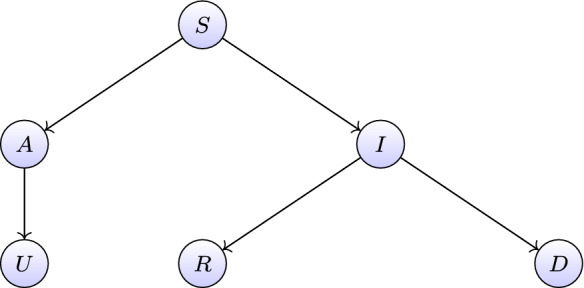
Compartmental model with a large set of asymptomatic infectives [32, 33]

This is why the nonlinear compartmental model describing a set of asymptomatic infectives proposed by Gaeta [32], and slightly revised by Paggi [33], is here adopted to simulate the infection nonlinear dynamics. The resulting compartmental model is a susceptible–infected–recovered–deceased model with a large set of asymptomatic infectives (A-SIRD), in which susceptible individuals *S*(*t*) can evolve in one of the two classes of infected and infective people, namely symptomatic *I*(*t*) or asymptomatic *A*(*t*) individuals. People can thus move from the compartment of symptomatic individuals into two other compartments, that of registered recovered *R*(*t*) or that of deceased *D*(*t*). People in the compartment of asymptomatic individuals can be removed and pass to the compartment of unregistered recovered *U*(*t*), which collects individuals passing unnoticed through the infection. The overall model is illustrated in Fig. 13.

By omitting the dependence from the time variable *t*, the numerical model here employed to simulate the infection dynamics is based on the following set of nonlinear ordinary differential equations:

4 $$\begin{aligned} \begin{aligned} \dfrac{\text {d}S}{\text {d}t}&=-\beta (I+A)S, \\ \dfrac{\text {d}I}{\text {d}t}&=\xi \beta (I+A)S-\gamma I-\mu I , \\ \dfrac{\text {d}A}{\text {d}t}&=(1-\xi )\beta (I+A)S-\eta A , \\ \dfrac{\text {d}R}{\text {d}t}&=\gamma I , \\ \dfrac{\text {d}D}{\text {d}t}&=\mu I , \\ \dfrac{\text {d}U}{\text {d}t}&=\eta A . \end{aligned} \end{aligned}$$

Note that the subset of variables $\mathbf{x}=(S, I, A)$ acts as master variables, while the subset $\mathbf{y}=(R, D, U)$ represents the slave variables. The state vector $\mathbf{z}=(S,I,A,R,D,U)$ is thus partitioned as $\mathbf{z}=[\mathbf{x}|\mathbf{y} ]$. The master and slave state equations can be expressed, respectively, as

5 $$\begin{aligned} \dot{\mathbf{x}}&=\mathbf{f}(\mathbf{x};\varvec{\theta }), \end{aligned}$$

6 $$\begin{aligned} \dot{\mathbf{y}}&=\mathbf{g}(\mathbf{x};\varvec{\theta }), \end{aligned}$$

where $f_1=-\beta x_1 (x_2+x_3),\,\, f_2=\xi \beta x_1 (x_2+x_3) -(\gamma +\mu )x_2,\,\, f_3=-\eta x_3+(1-\xi )\beta x_1 (x_2+x_3)$ and $g_1=\gamma x_2,\,\,\, g_2=\mu x_2, \,\,\,g_3=\eta x_3$ and $\varvec{\theta }=(\beta ,\gamma ,\mu ,\eta ,\xi )$ is the vector collecting the model parameters regulating the infection dynamics. In particular, $\beta$ represents the contact rate, $\xi$ denotes the probability that an individual who gets infected passes to the class of symptomatic infected people, $\gamma$ regulates the rate of growth of the recovered, $\mu$ dictates the rate of change of the deceased, and $\eta$ the rate of change of the unregistered. Once Eq. (5) is solved for $\mathbf{x}$, the master solution is substituted into Eq. (6) and solved to yield $\mathbf{y}$. An asymptotic treatment of these equations can yield closed-form approximate solutions for parametric investigations. This is part of ongoing research.

Note that the above equations satisfy the following conservation relationship:

7 $$\begin{aligned} \sum _{i=1}^6 {\dot{z}}_i=0\, \end{aligned}$$

which stems from the fact that the compartmented population is given by $P= \sum _{i=1}^6 x_i=S+I+A+D+R+U$, and a conservation law is assumed, namely ${\dot{P}}=0$.

The linear solution for the master state variables, upon assuming constant parameters $(\gamma , \mu ,\eta )$, is given by $(S,I,A)=(S_0,I_0 e^{-(\gamma +\mu )t},A_0 e^{-\eta t}).$ Hence, the eigensolutions can be expressed as

8 $$\begin{aligned} \mathbf{u}(t)=(c_1,c_2e^{-(\gamma +\mu )t}, c_3 e^{-\eta t}), \end{aligned}$$

where the coefficients $c_1$, $c_2$, $c_3$ can be determined from the initial conditions.

It is important to highlight that this compartmental model proposed by Gaeta [32] is based on the assumption that both symptomatic and asymptomatic infected people are infective in the same way. From this standpoint, an interesting feature of the model is related to the physically grounded parameter $\xi \in [0,\,1]$ representing the probability that an infected individual becomes symptomatic infected. Hence, $1-\xi$ is the probability that an individual who gets infected passes to the class of asymptomatic infected people.

In order to take into account the effects of the national lockdown on epidemic dynamics, a piecewise constant $\beta$ is herein introduced following [32]. In detail, it is here assumed that $\beta$ varies according to:

9 $$\begin{aligned} \beta = \left\{ \begin{array}{l l} \beta _0 &{} \text{ if } t < T_1 + \tau _1\\ \beta _0 \rho _1 &{} \text{ if } T_1 + \tau _1 \le t \le T_2 + \tau _2\\ \left( \beta _0 \rho _1 \right) \rho _2 &{} \text{ if } t > T_2 + \tau _2\\ \end{array} \right. , \end{aligned}$$

where $\beta _0$ is the initial value of $\beta$ (i.e., the value of $\beta$ not affected by lockdown measures), which reduces to $\beta _0 \rho _1$ and further to $\left( \beta _0 \rho _1 \right) \rho _2 = \beta _0 \rho$ (i.e., $\rho =\rho _1\rho _2$) when increasingly stricter national lockdown measures are enforced at $T_1$ and $T_2$, respectively. Here, $T_1$ and $T_2$ indicate March 8, 2020, and March 22, 2020, respectively (see Table 1). The time delays $\tau _1$ and $\tau _2$ reflect the delay between the lockdown enforcement at time $T_1$ and $T_2$, respectively, and the appearance of its effects on the epidemic dynamics. Differently from [32, 33], $\gamma$ and $\mu$ are not considered as constant values, but they are assumed as linearly time-varying parameters according to:

10 $$\begin{aligned} \gamma= & {} \max \{ a_\gamma + b_\gamma t,0 \}, \end{aligned}$$

11 $$\begin{aligned} \mu= & {} \max \{ a_\mu + b_\mu t,0 \}. \end{aligned}$$

Conversely, the parameters $\eta$ and $\xi$ are assumed to be time-invariant.

#### Parametric identification of the compartmental model

The parametric identification of the model given by Eq. (4) is pursued to provide further insights about the COVID-19 pandemic time- and space-wise evolution, at both regional and national levels, rather than assessing the predictive capability of the model itself. To this end, the parametric identification of the infection nonlinear dynamic model is performed according to Quaranta et al. [49]. Specifically, a differential evolution algorithm is implemented in order to minimize the sum of the normalized mean squared errors between available epidemic data and corresponding numerical results. Therefore, the following objective function is sought to be minimized:

12 $$\begin{aligned} \begin{aligned} f (\varvec{\theta }) =&\,w_I\dfrac{1}{L\sigma ^2_{{\bar{I}}}}\sum _{k=1}^L{({\bar{I}}_k-I_k(\varvec{\theta }))^2} \\&+w_D\dfrac{1}{L\sigma ^2_{{\bar{D}}}}\sum _{k=1}^L{({\bar{D}}_k-D_k(\varvec{\theta }))^2} \\&+w_R\dfrac{1}{L\sigma ^2_{{\bar{R}}}}\sum _{k=1}^L{({\bar{R}}_k-R_k(\varvec{\theta }))^2}, \end{aligned} \end{aligned}$$

where ${\bar{I}}_k$, ${\bar{D}}_k$ and ${\bar{R}}_k$ are the epidemic data available at the *k*th day, whereas $I_k$, $D_k$, and $R_k$ are the corresponding numerical results obtained for a given set of model parameter vector $\varvec{\theta }$. Moreover, *L* is the time window employed for the parametric identification (i.e., number of days considered for the analysis), while $\sigma ^2_{{\bar{I}}}$, $\sigma ^2_{{\bar{D}}}$ and $\sigma ^2_{{\bar{R}}}$ are the variances of the samples $$\begin{Bmatrix}{\bar{I}}_1 \ldots {\bar{I}}_L\end{Bmatrix}$$, $$\begin{Bmatrix}{\bar{D}}_1 \ldots {\bar{D}}_L\end{Bmatrix}$$ and $$\begin{Bmatrix}{\bar{R}}_1 \ldots {\bar{R}}_L\end{Bmatrix}$$, respectively. Finally, $w_I$, $w_D$ and $w_R$ are suitable weighting factors for the different error measures.

#### Results of the compartmental model for the regional and national scales

From a computational standpoint, the master state equations given by Eq. (4) are solved for *S*, *I*, and *A*. To this end, the classical Dormand–Prince method belonging to the Runge–Kutta family of solvers is used by imposing suitable initial conditions. On the other hand, the slave equations given by Eqs. (4)$_4$ and (4)$_5$ are taken into account, through the corresponding integral form, to calculate *R* and *D* once *I* is estimated, and the involved constants are determined employing the initial conditions. For such a task, the classical trapezoidal numerical integration is employed. Initial conditions are established by assuming $S_1=P$ (where *P* is the population, the number of regional or national residents) and $A_1=(1-\xi )I_1/\xi$ according to [32], whereas $I_1$, $R_1$, and $D_1$ are defined on the basis of the available data. It is noted that the solution of Eq. (4)$_6$ for *U* is not necessary for the present study, and thus it is omitted.

The differential evolution algorithm employed for minimizing $f (\varvec{\theta })$ in Eq. (12) implements a cur-to-best/1 mutation scheme with scale factors equal to 0.50, and a binomial crossover according to a crossover rate equal to 0.50. The parameter population size is equal to 30, whereas the procedure stops once 50 iterations are completed. The parametric identification is performed for a time window running from the first day in which at least ten positive cases are recorded, up to the last day in the available database at the time when the presented analyses were carried out, i.e., May 12, 2020. Although this implies considering 8 days beyond the relaxation of lockdown measures, it is assumed that the overall compartmental model described by Eqs. (4)–(11) still holds true during this short time overhang.

The search space has been carefully defined according to available information and medical evidences. Based on the preliminary estimate by Gaeta [32], the optimal value of $\beta _0$ at the national level is searched between the values $1.0 \cdot 10^{-9}$ and $1.0 \cdot 10^{-8}$, whereas a quite large interval spanning from $1.0 \cdot 10^{-8}$ to $3.0 \cdot 10^{-7}$ is considered to look for the optimal value of $\beta _0$ at the regional level. Daily samples $$\begin{Bmatrix}{\bar{\gamma }}_1 \ldots {\bar{\gamma }}_L\end{Bmatrix}$$ and $$\begin{Bmatrix}{\bar{\mu }}_1 \ldots {\bar{\mu }}_L\end{Bmatrix}$$ have been calculated as the ratio between the daily rate of recovered or deceased individuals by the daily number of symptomatic infected according to Eq. (4)$_4$ and Eq. (4)$_5$, respectively. Hence, the search space for $(a_\gamma ,b_\gamma )$ and $(a_\mu ,b_\mu )$ is defined as $\pm 20\%$ of the estimates carried out by performing the linear regression of the samples $$\begin{Bmatrix}{\bar{\gamma }}_1 \ldots {\bar{\gamma }}_L\end{Bmatrix}$$ and $$\begin{Bmatrix}{\bar{\mu }}_1 \ldots {\bar{\mu }}_L\end{Bmatrix}$$, respectively.

On the other hand, it is presumed that the optimal (time-invariant) value of $\eta$ should be related, to some extent, to that of $\gamma$. Hence, the optimal value of $\eta$ is assumed to be comprised between $10^{-1}$ and ten times the average value of the samples $$\begin{Bmatrix}{\bar{\gamma }}_1 \ldots {\bar{\gamma }}_L\end{Bmatrix}$$. Based on the available medical information, the incubation time for COVID-19 conservatively varies between 2 days and 14 days, which is therefore assumed as the time interval for searching the optimal value of $\tau _1$ and $\tau _2$. Moreover, $\rho _1$ and $\rho _2$ are both constrained to vary between $10^{-2}$ and 1. The search of the optimal value of $\xi$ is performed between 0.05 and 0.20, taking into account the preliminary estimate provided by Gaeta [32]. Under the assumption that *R* and *D* are estimated directly from *I*, the differential evolution algorithm is forced to prioritize the minimization of the normalized mean squared error in terms of symptomatic infected people, and thus it is assumed $w_I=1$, whereas $w_D=w_R=1/3$.

**Fig. 14:**
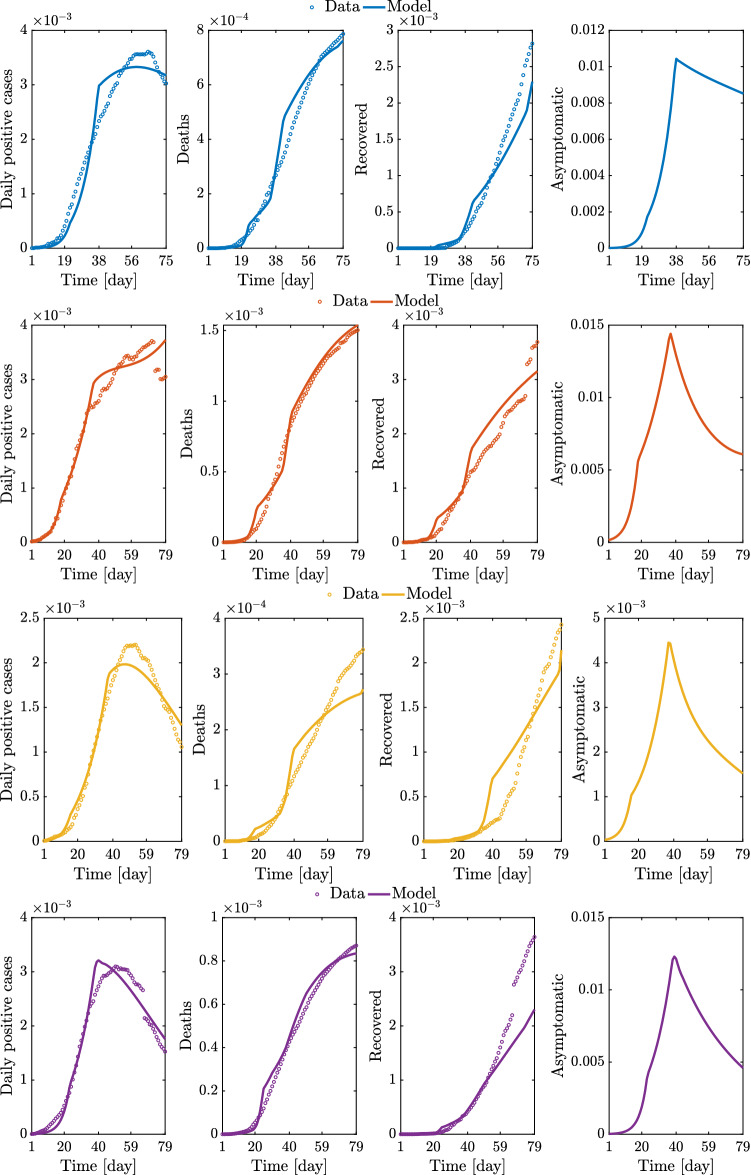
Comparison between data and model simulations (normalized with the number of residents) for Piemonte, Lombardia, Veneto, and Emilia-Romagna considering a time window running from the first day in which at least ten positive cases are recorded (day 1) up to May 12, 2020

**Fig. 15:**
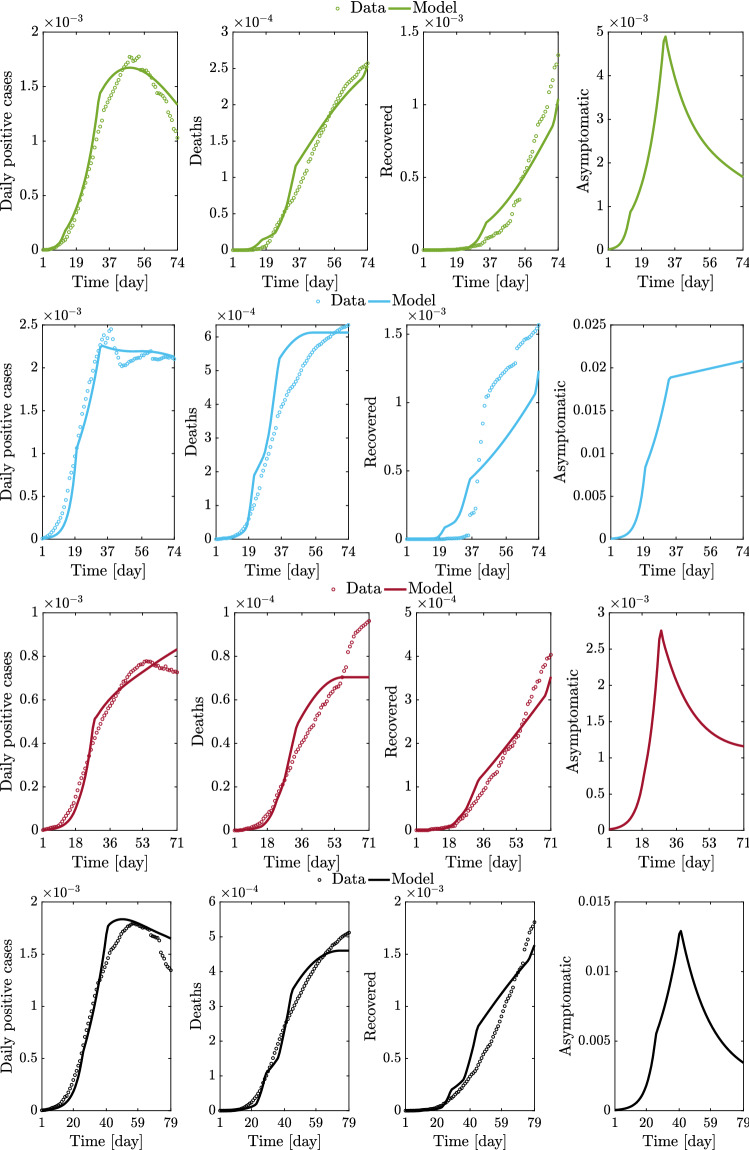
Comparison between data and model simulations (normalized with the number of residents) for Toscana, Marche, Lazio, and Italy considering a time window running from the first day in which at least ten positive cases are recorded (day 1) until May 12, 2020

**Fig. 16:**
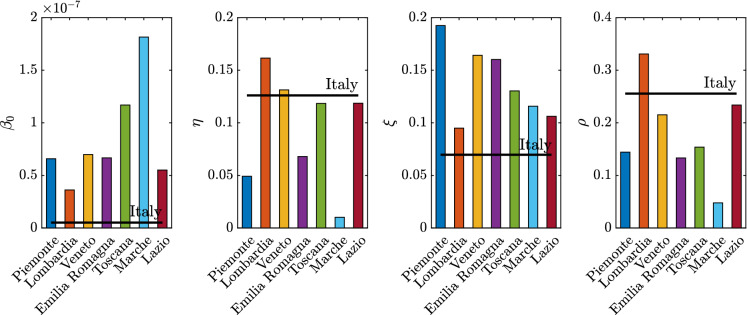
Identified model parameters for the selected regions and for Italy

**Fig. 17:**
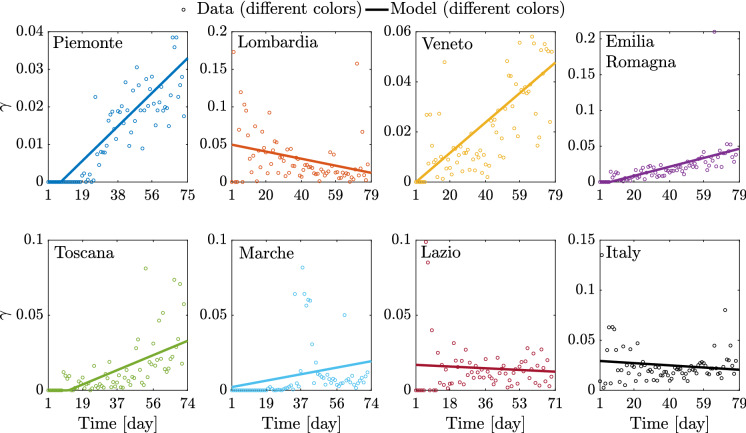
Comparison between data and identified linearly time-varying parameter $\gamma$ for the selected regions and the whole country considering a time window running from the first day in which at least ten positive cases are recorded (day 1) until May 12, 2020

**Fig. 18:**
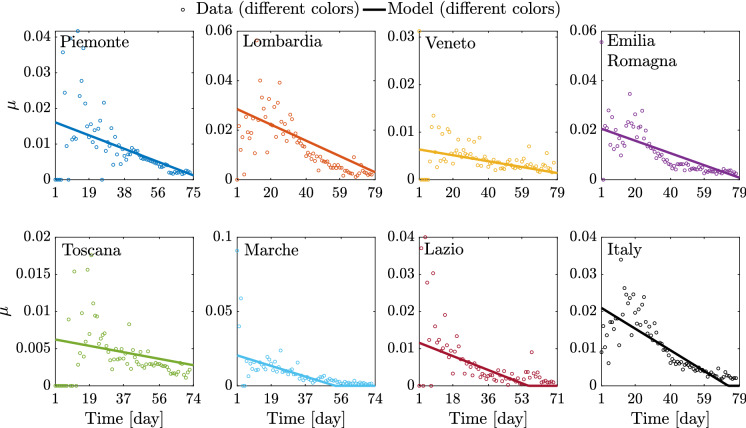
Comparison between data and identified linearly time-varying parameter $\mu$ for the selected regions and the whole country considering a time window running from the first day in which at least ten positive cases are recorded (day 1) until May 12, 2020

The full set of results for the regional and national epidemic evolutions is provided in Figs. 14, 15, 16, 17, and 18.

Overall, these outcomes provide a further insight into the dynamic evolution of the COVID-19 pandemic in Italy and highlight once again how it evolved at different territorial scales. After the parametric identification, it can be observed that the compartmental model provides a reasonable approximation of the number of daily positive cases, deceased, and recovered people over time, especially within one month after the occurrence of ten positive cases. The number of daily positive cases in the vicinity of the peak and beyond, as well as the day of peak attainment, is reasonably well approximated for those regions in which enough data in the descending branch are available within the considered time window (as is the case with Veneto, Emilia-Romagna, Toscana, and Marche). Otherwise, the accuracy level reduces, or the peak itself is not achieved yet (as is the case with Piemonte, Lombardia, Lazio, and Italy). In general, it can also be noted that the estimated number of deceased and recovered people at the end of the considered time window is slightly underestimated. From the obtained results at the regional level, it is estimated that the number of people being infected without showing symptoms has attained a daily peak between 0.28 and 2.08% of the number of residents, whereas the corresponding national value is 1.29%. The daily peak of asymptomatic people at national level is very similar to that recorded in some of the most severely affected (and most populated) regions in Italy, namely Piemonte, Lombardia, and Emilia-Romagna. It is interesting to note that these three regions in northern Italy exhibit a comparable daily peak of asymptomatic people, around 1% of the number of residents, as well as a similar value of the ratio between the peaks of daily asymptomatic people and daily positive cases, which turns out to be larger than 3. In this context, the situation in Veneto seems rather different from that experienced by the other northern regions. Herein, the number of people that gets infected without showing symptoms has attained a daily peak equal to 0.44%, thereby becoming much lower than that reached in Piemonte, Lombardia, and Emilia-Romagna. Furthermore, the ratio between the peaks of daily asymptomatic people and daily positive cases in Veneto is close to 2, once again fairly lower than the value estimated for Piemonte, Lombardia, and Emilia-Romagna.

The possibility arises whether such differences between Piemonte, Lombardia, and Emilia-Romagna, on the one hand, and Veneto, on the other hand, might be explained by the fact that a more intensive tracing policy has been implemented in the latter as compared to the others, as already pointed out in Fig. 10. Among the seven considered regions, the maximum value of the ratio between the peaks of daily asymptomatic people and daily positive cases is attained in Marche, where it is close to 10. Since it has been recognized that undocumented infection facilitates the rapid dissemination of COVID-19, this high value might explain the fact that a large number of daily new positive cases have been recorded in Marche some days before this happened in other areas of Italy, as already noted in Fig. 7. At the national level, the ratio between the peaks of daily asymptomatic people and daily positive cases is estimated to be around 7. Altogether, these results seem to corroborate the possibility that the undocumented infected people can be 5-10 times the number of detected infected people.

Concerning the identified model parameters, an unbiased comparison of $\beta _0$ estimates requires a proper scaling with respect to the population size. Hence, once $\beta _0$ estimates at regional level are projected onto the whole Italian population, numerical values between $4.59 \cdot 10^{-9}$ and $7.23 \cdot 10^{-9}$ are obtained. The mean value among the considered regional estimates of $\beta _0$ is $5.51 \cdot 10^{-9}$ (the coefficient of variation is 16.61%), and it is close to the estimate obtained for the whole country, which is $5.14 \cdot 10^{-9}$. These values for $\beta _0$ are somewhat close to the previous national estimate of $3.77 \cdot 10^{-9}$ adopted in [32]. Additionally, it is found that the regional values of $\xi$ vary between 9.50 and 19.50%. The mean value among the considered regional estimates of $\xi$ is 13.81% (the coefficient of variation is 26.18%), and it is about twice as large as the corresponding estimate obtained at the national level, which is 6.97%. The significant scattering is basically due to the uncertainty inherent in the assessment of $\xi$, whose value yet is in fairly good agreement with the estimate of 10% adopted in [32].

**Fig. 19:**
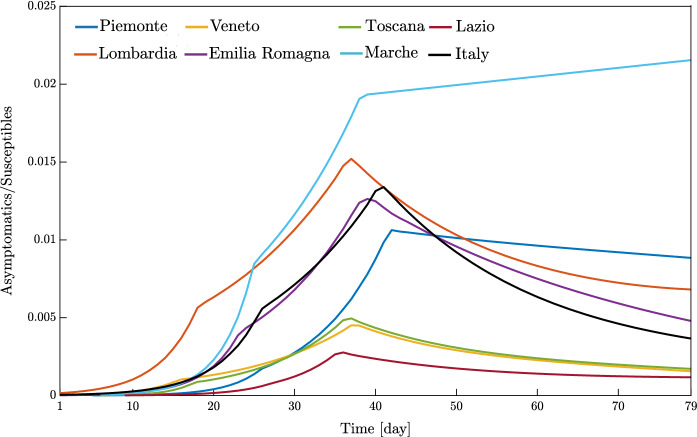
Trend of predicted asymptomatic cases (rescaled by the number of susceptibles) for the seven selected Italian regions and for Italy considering a time window running from the first day in which at least ten positive cases are recorded until May 12, 2020

Significant variations are also observed in terms of $\rho$ among the considered regions. In fact, regional values of $\rho$ between 4.77 and 33.08% are estimated. The mean value among the considered regional estimates of $\rho$ is 17.99% (the coefficient of variation is 50.00%), and it is rather close to the corresponding estimate obtained at the national scale, which is 25.55%. These final estimates are slightly larger than the reductive coefficient for $\beta _0$ in [32], in which $\rho$ is taken equal to 15%. Both data and model suggest that $\gamma$, i.e., the ratio between daily rate of recovered individuals by the daily number of symptomatic infected, grows in time in most regions, a circumstance that can be ascribed to the fact that COVID-19 diagnoses became faster over time than the early-stage detection. While this ratio can be almost constant in some cases, an opposite trend is observed in Lombardia. This might be due to the fact that the maximum capacity of intensive and semi-intensive care units was nearly attained in this region during the emergency phase. Both data and model also suggest that $\mu$, i.e., the ratio between daily rate of deceased individuals by the daily number of symptomatic infected, reduces in time, everywhere. This is attributable to diagnoses that have become more and more timely as well as to medical treatments that become more and more effective, as time goes by. Finally, the average regional delay for the lockdown measure to be effective has been estimated to be about 8.95 days (the coefficient of variation is 31.33%).

The virtue of the employed A-SIRD model is that it allows to predict an important epidemiological variable, namely the asymptomatic cases scaled by the number of susceptibles within the considered region or the country (see Fig. 19). Indeed, the number of asymptomatic cases plays a crucial role in the epidemic dynamics. The results show that Lombardia had the fastest growth of asymptomatic cases and most of the regions reached the peak around the second lockdown except for Marche (increasing trend past the second lockdown) and Piemonte (a very mildly decreasing trend past the second lockdown). Therefore, the second (strict) lockdown had a large impact on the main driving variable *I* and, in turn, on the other meaningful variable *A*, thus explaining the large slowdown of the epidemic spreading.

**Fig. 20:**
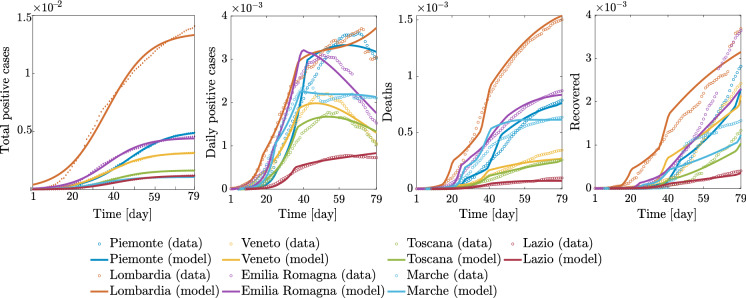
Comparison between data and model simulations (normalized with the number of residents) for the seven selected Italian regions considering a time window running from the first day in which at least ten positive cases are recorded until May 12, 2020

**Fig. 21:**
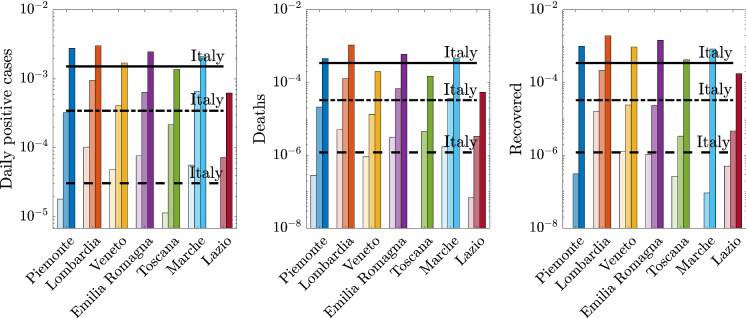
Average values of daily positive cases, deaths, and recovered for the relevant time windows: (i) from the first day in which at least ten positive cases are recorded up to March 7, 2020 (light color: regional values, dashed line: national value); (ii) from March 8, 2020, to March 21, 2020 (medium color: regional values, dash-dot line: national value); and (iii) from March 22, 2020, to May 12, 2020 (dark color: regional values, solid line: national value). (Color figure online)

Moreover, neglecting the presence of the large set of asymptomatic infectives can also lead to poor estimates of the basic reproduction number [12] at later stages of the epidemic development as shown in [16]. The comparison carried out in [16] between the *apriori* predictions and the *aposteriori* estimates based on field data which arise from the A-SIR model with those obtained using the standard SIR theory was shown to lead to erroneous estimates of the reproduction number. This uncertainty about the estimates of the basic reproduction number, in cascade, can lead to wrong decision-making policies to control the epidemic.

## How does the local spatial scale contribute to the global epidemic dynamics?

The analysis carried out in terms of the various epidemiological variables over time together with the identifications obtained using the logistic or the A-SIRD models shows clearly a picture of epidemic spreading which is rather nonuniform across the local provincial and regional scales (see Figs. 3, 4, 5, 6). This is further portrayed in Fig. 20, which shows the total positive cases, daily new cases, deceased, and recovered (normalized with respect to the residents) for the seven considered regions together with the logistic and A-SIRD model identifications. To quantify the differences in a concise way, the averages of daily new cases, deceased, and recovered are computed over the three meaningful time windows, namely $[t_0, T_1 - 1],$ $[T_1,T_2-1]$, and $[T_2, t_f],$ where $t_0$ indicates the time when at least ten positive cases are recorded, $T_1$ and $T_2$ correspond to the days elapsed from $t_0$ up to the first and second lockdowns, respectively, and $t_f$ is the 79th day after $t_0.$ These three averages for each variable are shown in the bar charts of Fig. 21 together with the average values for Italy.

**Fig. 22:**
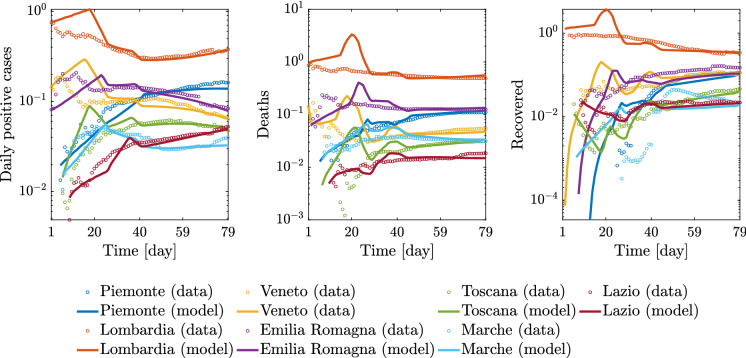
Comparison between data and model simulations for the examined regions (normalized with respect to the corresponding data and model simulations at national level) within a time window running from February 24, 2020 (day 1), up to May 12, 2020 (day 79)

The obtained results show that Lombardia is characterized by the fastest growing epidemic spreading and the most significant density of total positive cases with respect to its own population. Emilia-Romagna, Piemonte, and Veneto are well spaced from Lombardia. Even more spaced are the central regions of Toscana, Marche, and Lazio. Different similarities between regional epidemic spreading dynamics can be observed when we account for the daily positive cases, deceased, and recovered. The curves for the daily positive cases and the averages highlight the presence of at least three different areas (here and henceforth referred to as clusters) characterized by similar densities of cases: cluster A comprises Lombardia and Emilia-Romagna since they are both above the national averages across the three time windows, cluster B includes Piemonte and Marche, cluster C includes Toscana, which exhibits average values below the national averages at all times, and Lazio which turns out to be the less affected region, finally, cluster D comprises Veneto and Italy and is meant to describe the average dynamics. A different standpoint can be adopted to carry out the above regional-scale dynamic analysis by taking into account the daily cases normalized with respect to the overall number of positive cases in Italy leading to the assessment of some sort of regional weight factor in the national pandemic. This outcome can be appreciated in Fig. 22, which shows that the regions contribute differently to the national epidemic dynamics since Lombardia stands out as the primary regional scale (cluster $\hbox {A}^*$), followed by Emilia-Romagna, and Piemonte (cluster $\hbox {B}^*$), Marche and Toscana (cluster $\hbox {C}^*$), Lazio (cluster $\hbox {D}^*$), and finally, Veneto and Italy (cluster $\hbox {E}^*$). This assessment ensues from considering the first two time windows.

## Hierarchical clustering and multidimensional scaling of regional versus national epidemic dynamics

We employ a data-driven approach making use of several computational tools and visualization techniques to further identify, recognize, and quantify the very different epidemic evolutions at different geographical scales. The procedure comprises three main phases: (i) data items are compared in the perspective of employing suitable similarity (or dissimilarity) indices, (ii) a computational scheme is employed to process the obtained results toward visualization, and (iii) the obtained results are interpreted.

**Fig. 23:**
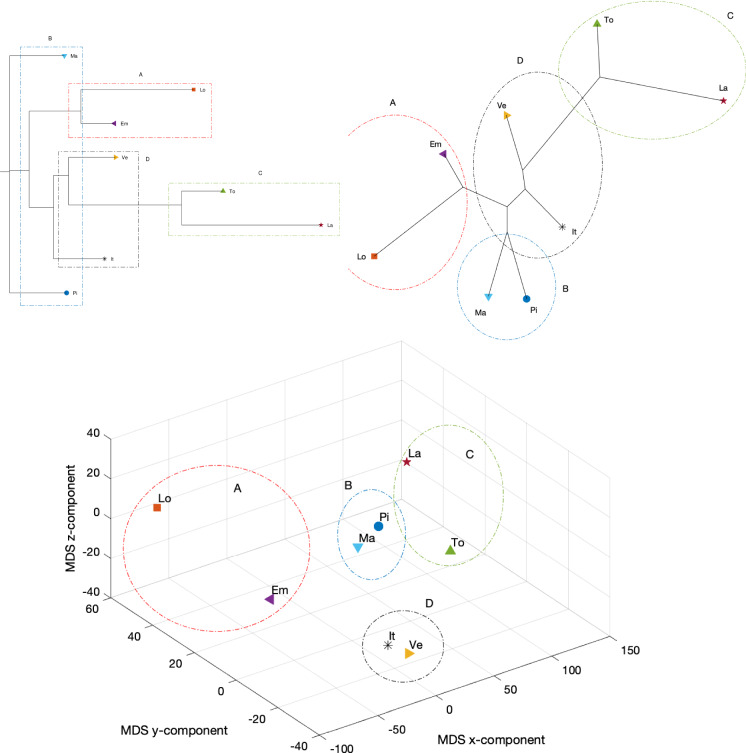
Dendrogram (top left), hierarchical tree (top right), and 3D multidimensional scaling plots (bottom) of Piemonte, Lombardia, Veneto, Emilia-Romagna, Toscana, Marche, Lazio, and Italy, using the Neighbor linkage criterion, for a single time window ($n_w=1$) of $T=70$ days using the Canberra distance

**Fig. 24:**
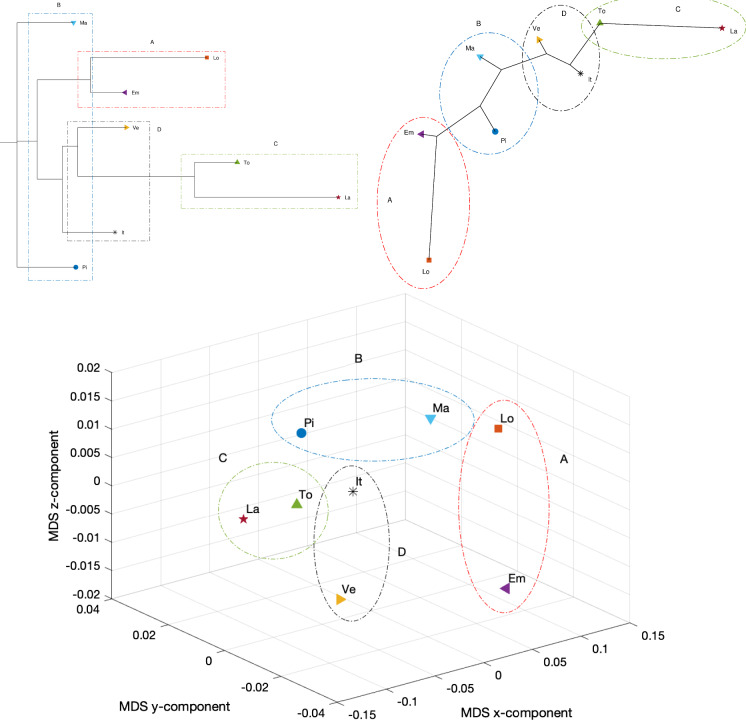
Dendrogram (top left), hierarchical tree (top right), and 3D multidimensional scaling plots (bottom) of Piemonte, Lombardia, Veneto, Emilia-Romagna, Toscana, Marche, Lazio, and Italy, using the Neighbor linkage criterion, for a single time window ($n_w=1$) of $T=70$ days using the Manhattan distance

**Fig. 25:**
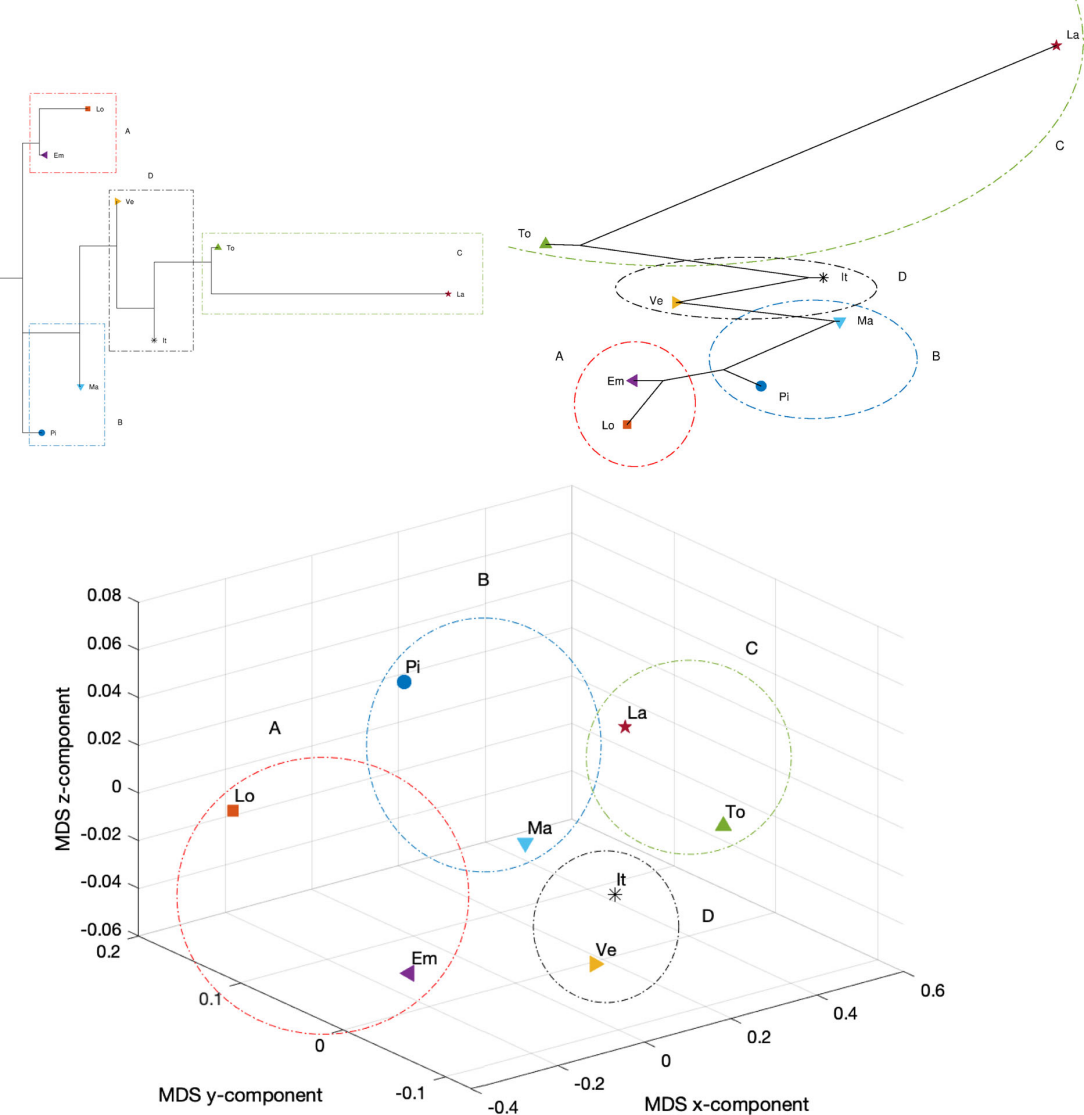
Dendrogram (top left), hierarchical tree (top right), and 3D multidimensional scaling plots (bottom) of Piemonte, Lombardia, Veneto, Emilia-Romagna, Toscana, Marche, Lazio, and Italy, using the Neighbor linkage criterion, for a single time window ($n_w=1$) of $T=70$ days using the Jaccard distance

**Fig. 26:**
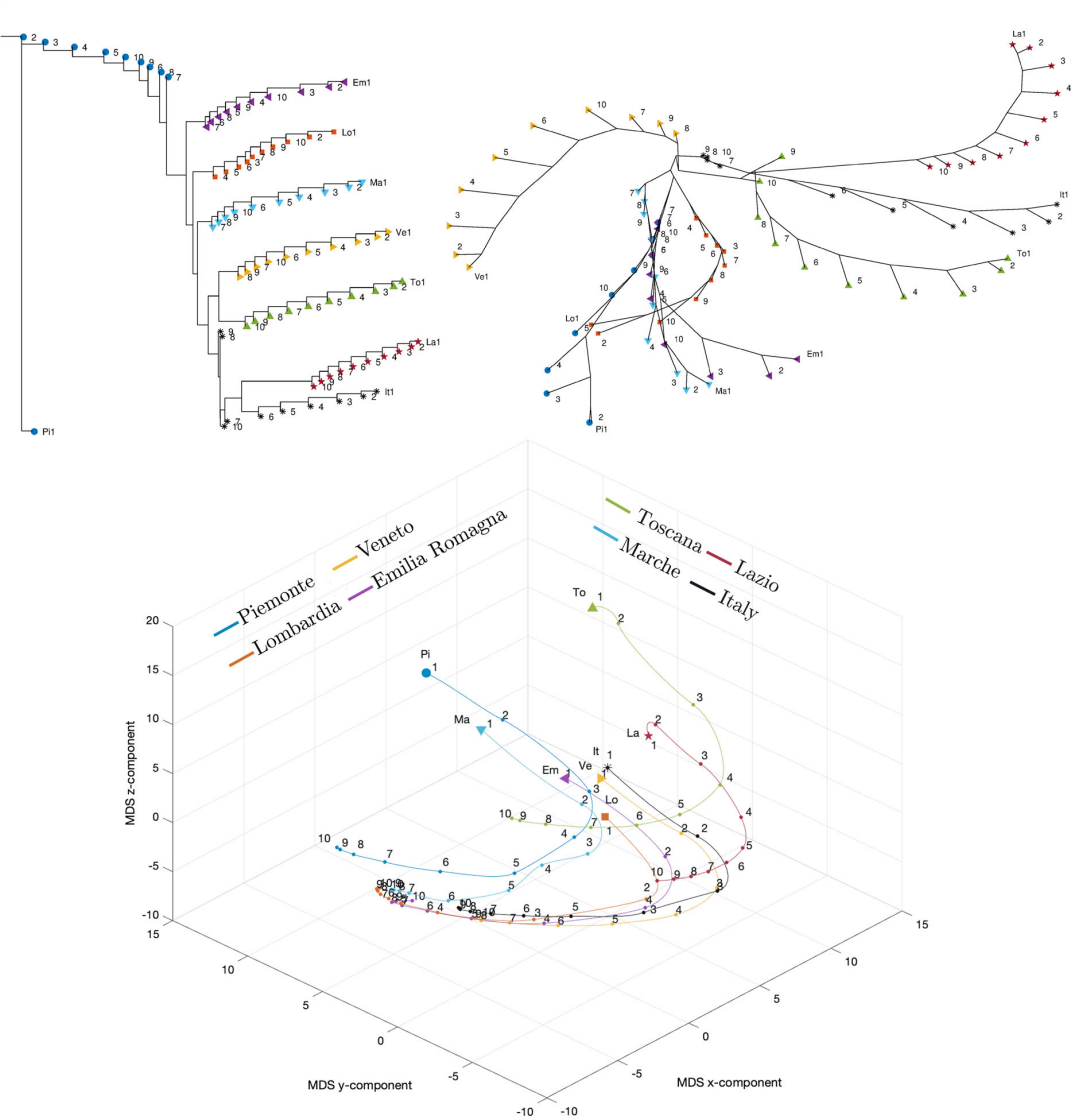
Dendrogram (top left), hierarchical tree (top right), and 3D multidimensional scaling plots (bottom) of Piemonte, Lombardia, Veneto, Emilia-Romagna, Toscana, Marche, Lazio, Italy for $n_w=10$ time windows of $T=7$ days using the Canberra distance

The first phase requires the definition of one or more criteria for comparing all items in the dataset. The concept of distance [41] is often adopted to quantify the dissimilarity between items expressed, e.g., as time series. A function *d* of two arguments/items, $x_i$ and $x_j$, can describe a distance if $d(x_i,x_j)\ge 0$ and satisfies three axioms [50]:

13 $$\begin{aligned} \begin{aligned} \text{ identity }: \, d(x_i,x_j)&= 0 \ \text{ if } \ x_i\ = x_j\\ \text{ symmetry }: \, d(x_i,x_j)&= d(x_j,x_i) \\ \text{ triangle } \text{ inequality } : \, d(x_i,x_j)&\le d(x_i,x_h) + d(x_h,x_j). \end{aligned} \end{aligned}$$

Our daily experience is mostly permeated with the notion of the Euclidean distance, which is a particular case of the Minkowski norm. However, a variety of different expressions have been proposed to compute distances, each one having pros and cons, and being more or less adapted to the characteristics of the processed data. The definition of distance can be generalized to encompass also nonsymmetric distances [51–56].

For the dataset under study, we employed the Canberra, Jaccard, Minkowski, Lorentzian, Arc-Cosine, and Divergence distances [57]. The Minkowski distance depends on one parameter and gives rise to the Manhattan, Euclidean, and Chebyshev distances as special cases. For the sake of parsimony, in the following we report only the results concerning the Canberra, Manhattan, and Jaccard distances between the *i*th and *j*th items $x_i$ and $x_j$ and expressed as

14 $$\begin{aligned} d_{C}\left( x_{i},x_{j}\right)= & {} {\displaystyle \sum _{k=1}^{3}}{\displaystyle \sum _{t=1}^{T}\frac{\left| x_{i,k}(t)-x_{j,k}(t)\right| }{x_{i,k}(t)+x_{j,k}(t)}}, \end{aligned}$$

15 $$\begin{aligned} d_{M}\left( x_{i},x_{j} \right)= & {} {\displaystyle \sum _{k=1}^{3}}{\displaystyle \sum _{t=1}^{T}\left| x_{i,k}(t)-x_{j,k}(t)\right| }, \end{aligned}$$

16 $$\begin{aligned} d_{J}\left( x_{i},x_{j}\right)= & {} {\displaystyle \sum _{k=1}^{3}\frac{{\displaystyle \sum \nolimits _{t=1}^{T}}\Big [x_{i,k}(t)-x_{j,k}(t)\Big ]^{2}}{{\displaystyle \sum \nolimits _{t=1}^{T}}\Big [x_{i,k}^{2}(t)+x_{j,k}^{2}(t)-x_{i,k}(t)x_{j,k}(t)\Big ]}}, \nonumber \\ \end{aligned}$$

where the first subscripts *i* and *j* indicate the data series for variable *x* related to region *i* and region *j*, respectively, and the second subscript *k* is the component of the state vector while the argument *t* denotes time. In our case, we consider three components, namely the daily positive cases *I*, recovered *R*, and deceased individuals *D* in each region. Furthermore, to avoid a bias toward regions with higher population, we normalize the values by dividing them by the total number of residents in each region.

**Fig. 27:**
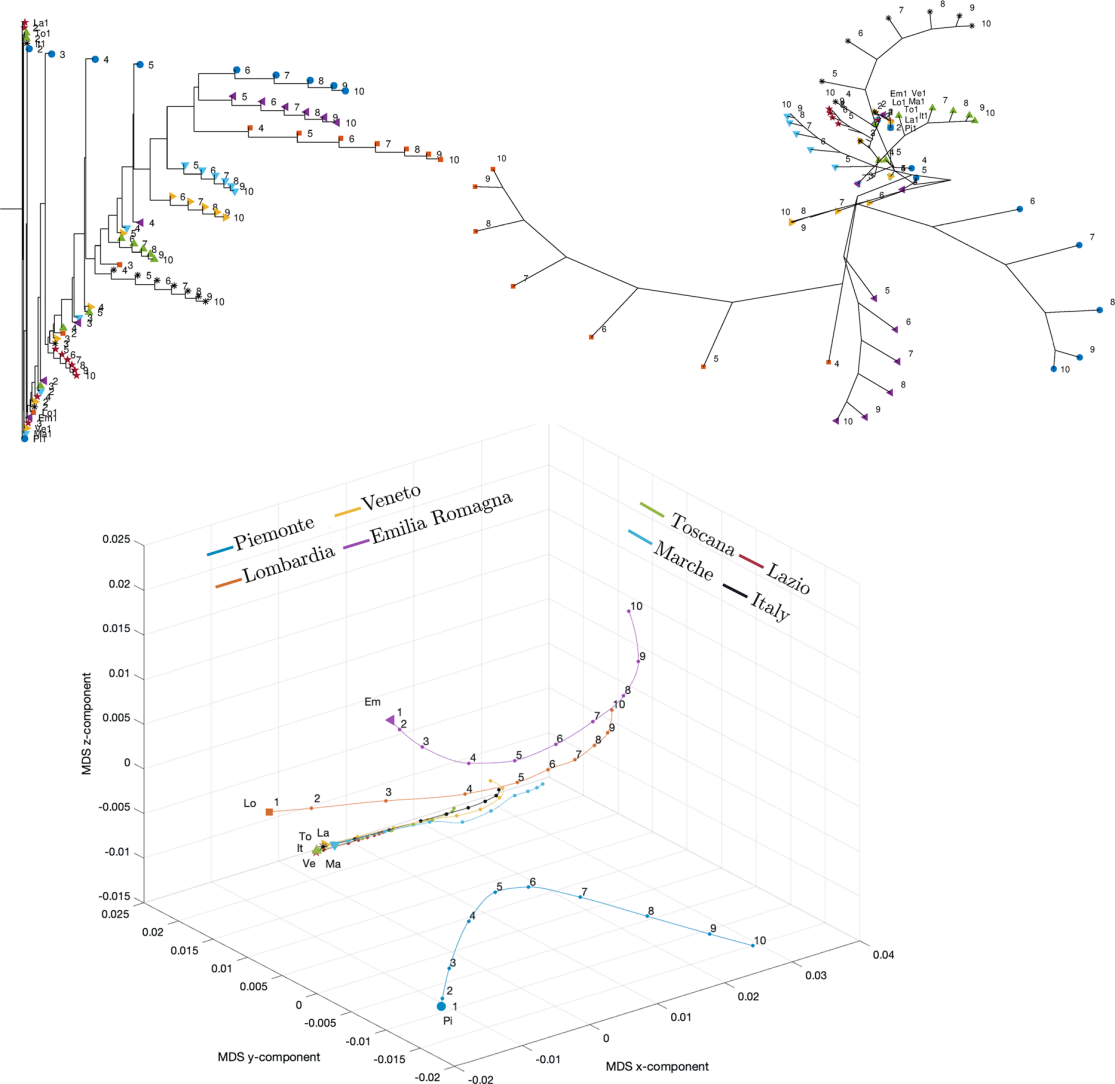
Dendrogram (top left), hierarchical tree (top right), and 3D multidimensional scaling plots (bottom) of Piemonte, Lombardia, Veneto, Emilia-Romagna, Toscana, Marche, Lazio, Italy for $n_w=10$ time windows of $T=7$ days using the Manhattan distance

**Fig. 28:**
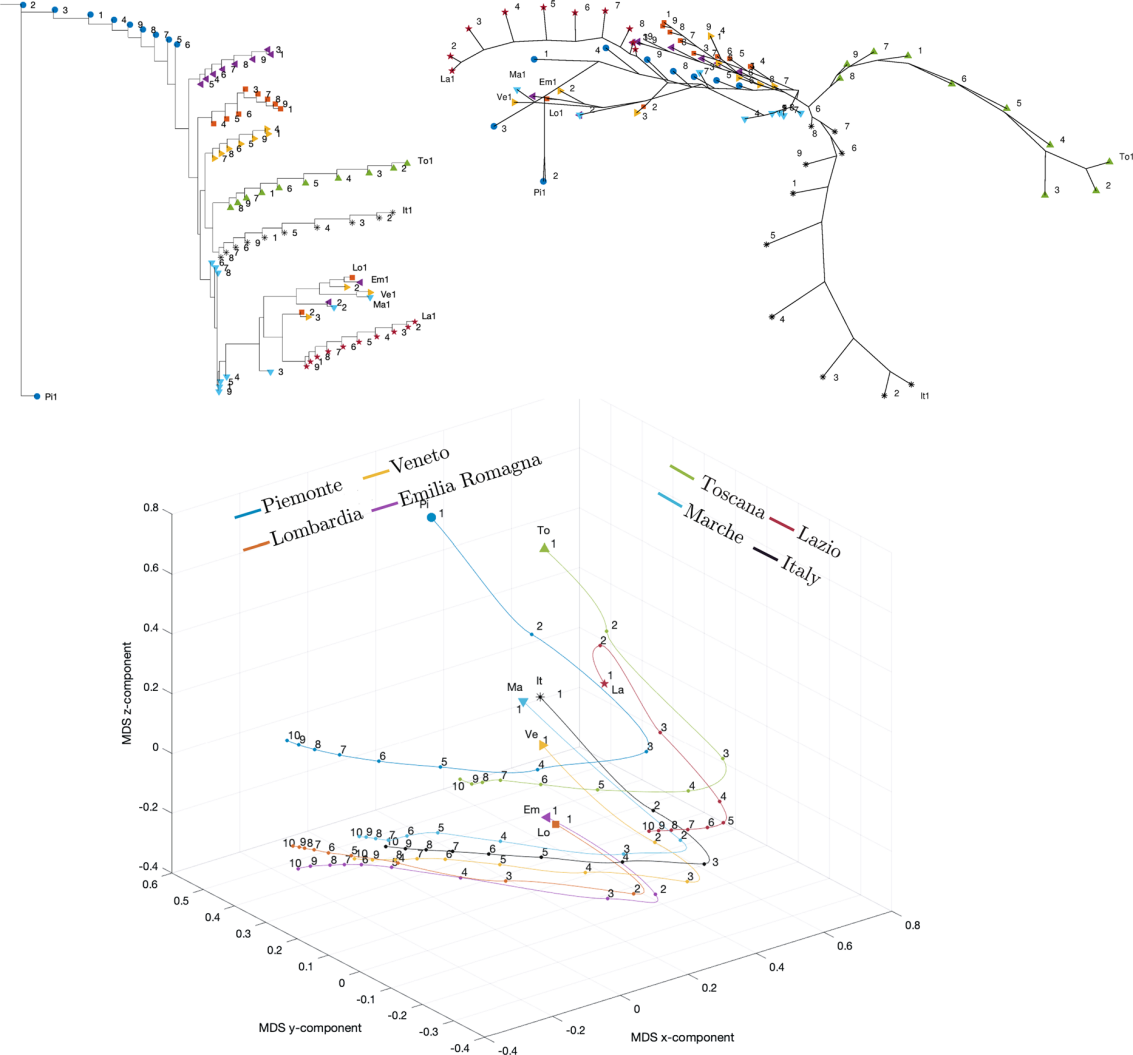
Dendrogram (top left), hierarchical tree (top right), and three-dimensional multidimensional scaling (bottom) representations of {Piemonte, Lombardia, Veneto, Emilia-Romagna, Toscana, Marche, Lazio, Italy} for $n_w=10$ time windows of $T=7$ days and the Jaccard distance

**Fig. 29:**
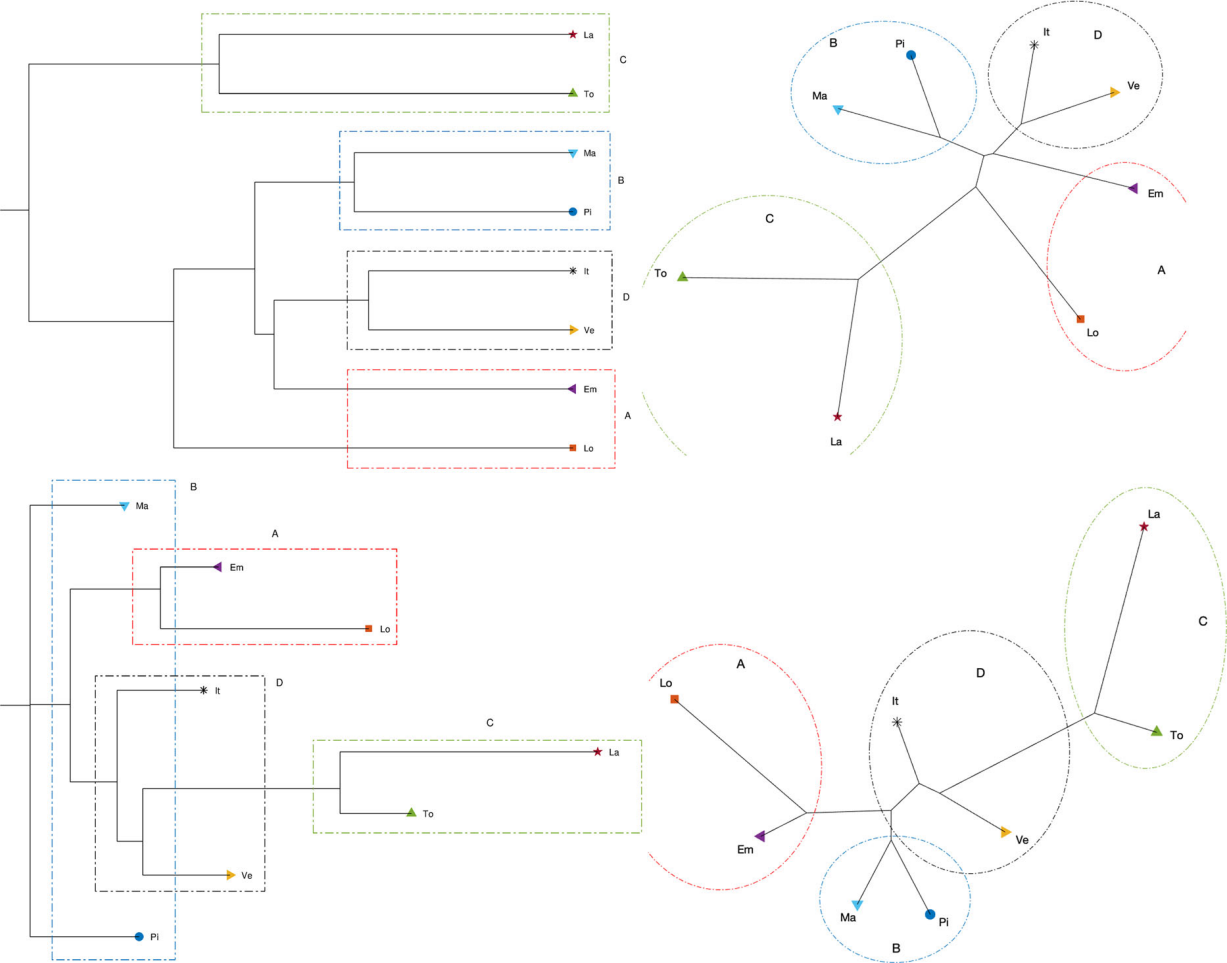
Dendrograms (left) and hierarchical trees (right) of Piemonte, Lombardia, Veneto, Emilia-Romagna, Toscana, Marche, Lazio, and Italy for a single time window ($n_w=1$) of $T=70$ days using the Canberra distance, and the Kitsch and Fitch linkage criteria (top and bottom, respectively)

**Fig. 30:**
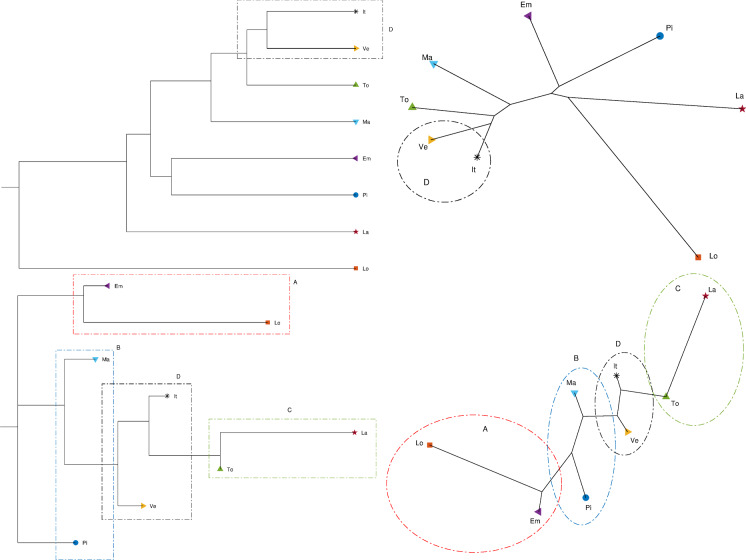
Dendrograms (left) and hierarchical trees (right) of Piemonte, Lombardia, Veneto, Emilia-Romagna, Toscana, Marche, Lazio, and Italy for a single time window ($n_w=1$) of $T=70$ days using the Manhattan distance, and the Kitsch and Fitch linkage criteria (top and bottom, respectively)

**Fig. 31:**
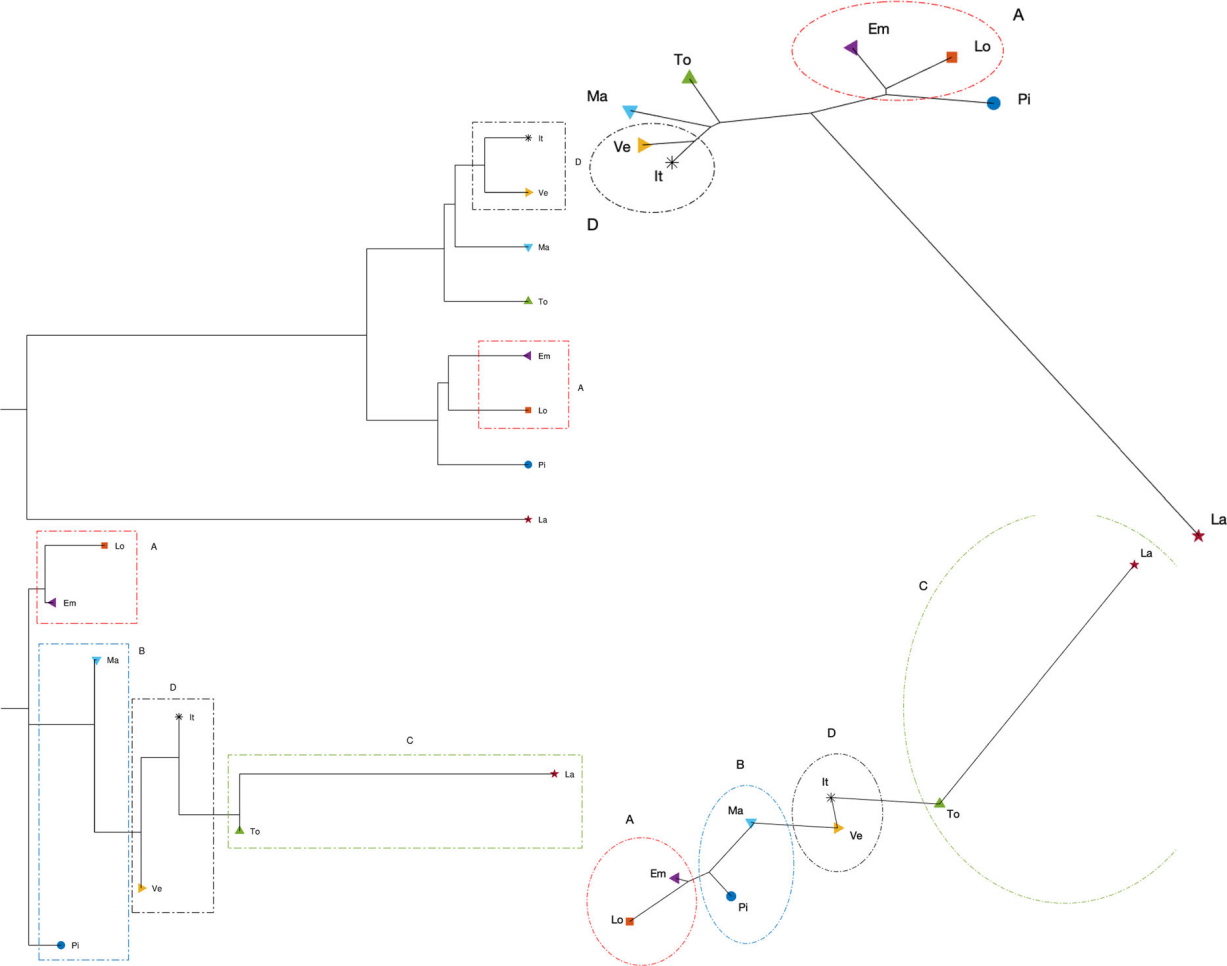
Dendrograms (left) and hierarchical trees (right) of Piemonte, Lombardia, Veneto, Emilia-Romagna, Toscana, Marche, Lazio, and Italy for a single time window ($n_w=1$) of $T=70$ days using the Jaccard distance, and the Kitsch and Fitch linkage criteria (top and bottom, respectively)

The Canberra distance can be interpreted as a weighted version of the Manhattan distance and is often used for data scattered around the origin. This metric enhances differences close to 0, where it is more sensitive to proportional than to absolute differences. On the other hand, it is less influenced than the Manhattan distance by variables with high values. The Jaccard distance is the ratio of the size of the symmetric difference (or disjunctive union) to the union of two sets of elements. The idea looks similar to that of Venn diagrams with $d_{J}=\frac{\left| A\cup B\right| -\left| A\cap B\right| }{\left| A\cup B\right| }$, with *A* and *B* denoting the two sets. The Jaccard distance is useful for comparing observations with categorical variables.

We consider the time window from March 1 to May 9, 2020, for two scenarios: (i) a single time window (i.e., $n_w=1$) of $T=70$ days, and (ii) $n_w=10$ time windows of $T=7$ days each (i.e., 10 weeks). Besides the regions Piemonte, Lombardia, Veneto, Emilia-Romagna, Toscana, Marche, and Lazio, the eighth component represents Italy.

In the case of $n_w=1$ the indices *i* and *j* refer to eight items and, therefore, $i,j=1,\ldots ,8$. Alternatively, if $n_w=10$, then the number of items is proportional to both the number of regions and time windows giving $i,j=1,\ldots ,80$.

In summary, we compare all vectors $x_{i,k}(t)$ and construct symmetric matrices with zeros in the main diagonal of item-to-item distances, $\varDelta _C=\left[ d_{C}\right]$, $\varDelta _M=\left[ d_{M}\right]$ and $\varDelta _J=\left[ d_{J}\right]$, that will be the source of information for further processing. Therefore, these matrices have size $8 \times 8$ and $80 \times 80$, respectively, for the time windows of $T=70$ and $T=7$ days, respectively.

The second phase consists of processing the information included in the matrices $\varDelta _C=\left[ d_{C}\right]$, $\varDelta _M=\left[ d_{M}\right]$, and $\varDelta _J=\left[ d_{J}\right]$. For visualization, within the large array of available techniques, we make use of dendrograms, hierarchical trees, and multidimensional scaling.

For the dendrograms and hierarchical trees, we adopt hierarchical clustering [28, 34–36, 58]. This computational technique arranges the items as elements of the matrices according to a graphical structure reflecting the distances between them in the perspective of the adopted clustering metric [59]. There are two algorithms, one called *agglomerative clustering* and the other *divisive clustering*. For both schemes, the numerical iterations follow a linkage criterion, based on the distances between pairs of items [60]. The clustering “quality” can be measured by the cophenetic correlation [61]. In the following, we adopt the Phylip set of programs (http://evolution.genetics.washington.edu/phylip.html), with the Neighbor linkage criterion and the Drawgram and Drawtree for constructing the dendrograms and hierarchical trees, respectively. For further details, interested readers can refer to [62, 63]. The interpretation of the “leafs” of the dendrograms or trees is the third phase and is based on their relative position with respect to the nodes and root.

The third visualization method consists of the multidimensional scaling. This is a technique for clustering and visualizing multidimensional data, using the same input matrix [64] for which, in this case, the items are represented by points. The computational iterations assign the points in a low-dimensional space, trying to reproduce the original distances from the higher dimensional space to which the items belong. We have also several possible criteria for the numerical optimization such as the Shepard and Stress diagrams.

The interpretation of the plots is the third phase and is based on the emerging clusters of points [65, 66]. The absolute coordinates of the points, the format of the clusters, and the axes do not have a physical meaning. We employ MATLAB (https://www.mathworks.com/products/matlab.html) for the computations, in particular the commands cmdscale and Sammon. For further details, see [37–40, 67].

Figures 23, 24, and 25 depict the dendrograms, hierarchical trees, and 3D multidimensional scaling, for a single time window of $T=70$ days, and the Canberra, Manhattan, and Jaccard distances, respectively. On the other hand, Figs. 26, 27, and 28 show the corresponding representations when considering $n_w = 10$ time windows of $T=7$ days each. The labels (Pi, Lo, Ve, Em, To, Ma, La, It) stand for the regions (Piemonte, Lombardia, Veneto, Toscana, Marche, Lazio, and Italy), respectively, while the indices $\left\{ 1,\ldots ,10\right\}$ indicate the progressive time windows.

In general, dendrograms are apparently easier to read, while the trees are easier to interpret besides exhibiting also an aesthetic appeal. The dendrograms require a reading from the root to the leafs (from left to right in the plots), and this may be misleading. The trees take a better advantage of the 2D space than the dendrograms, but lead often to confusing areas with overlapping labels. Moreover, both cases allow only a discrete representation of time in terms of time windows since it is rare to experience large jumps. The 3D MDS charts are harder to handle since they require the user to rotate the plot to find the best perspective. In the MDS plots of Figs. 26, 27, and 28, the spline connecting the items for a given region shows the time evolution. Contrary to the dendrograms and trees, the MDS plots allow the visualization of the time evolution.

In all cases, Italy is somewhere in the “middle” of the seven regions as one should expect, since it is a kind of weighted average of the 21 regions into which the country is divided. Also, when adopting a single time window, we observe in all plots the emergence of the same patterns although with a distinct graphical arrangement: a cluster formed by Lombardia and Emilia-Romagna denoted by A, a cluster made by Marche and Piemonte (denoted by B), a cluster encompassing Toscana and Lazio (marked as C), and an “average” cluster (denoted by D) which includes Veneto and Italy. As seen, none of the considered regions, except for Veneto, is quite close to the average epidemic evolution for Italy.

As mentioned before, the Canberra distance provides a distance in a relative perspective in contrast to the Manhattan distance that works in absolute terms. The Jaccard distance is a measure of how dissimilar two sets are. Therefore, depending on the metric, we can highlight distinct aspects of the data. The same type of dilemma occurs also with the construction of the dendrograms and hierarchical trees when adopting hierarchical clustering. Figures 29, 30, and 31 depict the dendrograms and hierarchical trees obtained by means of the Kitsch and Fitch linkage criteria. The ensuing representations show the same clustering when using the Canberra distance, while they do not exhibit a clear pattern when adopting the Kitsch linkage criterium with the Manhattan and Jaccard distances.

When adopting $n_w=10$ time windows spanning one week each, we verify large/small variations for the initial/final periods of analysis, showing very different initial transients, but some approaching a common dynamic pattern toward the end of the observation period.

As previously mentioned, the Canberra, Manhattan, and Jaccard distances have focused on distinct aspects of the dataset. In the case of dendrograms and trees, the Canberra distance yields slightly more discernible representations. For the 3D multiscaling representations, the Canberra and Jaccard distances give rise to plots of the same type, while the Manhattan distance highlights the dynamics of Piemonte, Lombardia, and Emilia-Romagna at the expenses of providing less distinction between the other four regions and Italy. Therefore, the best analysis strategy is confirmed to be based on the adoption of a number of distances so as to establish a trade-off between the pros and cons of each metric.

## Conclusions

This work tackled a multi-scale epidemic dynamic analysis of COVID-19 in Italy recognizing remarkably different evolutions at different geographical scales.

We adopted a variety of models and data-driven tools to support the analysis. In particular, the regional territorial scale (and partly the provincial/county scale) was selected as the local scale (the spatial domain occupied by Italy is divided into 21 regional subdomains of different sizes) with respect to the macronational scale, although the regional scale is closer to a mesoscale, whereas the truly local scale is represented by the order-of-magnitude smaller provinces (one-fifth of an average region) and towns (one-hundredth of an average region). This choice was dictated by the full availability of data.

The logistic model was employed to identify the so-called epidemic curves in terms of cumulative positive cases, across the seven selected regions where more than 80% of the cases were recorded. Notwithstanding the capability of the logistic regression in describing well the regional as well as national epidemic curves, this approach is clearly not suitable to show a true epidemic evolution which comprises different groups/compartments such as the susceptibles, infected, asymptomatic infectives, etc. To this end, we employed a modified A-SIRD model which accounts for the time evolution of the susceptibles, symptomatic, and asymptomatic infectives, registered or unregistered recovered, deceased individuals [33]. While this model proved to be reasonably accurate in describing the epidemic data in Italy in previous studies, we found out, via extensive parameter identification, that two of the governing parameters, namely $\gamma$ and $\mu$, cannot be considered time invariant across the whole epidemic evolution, but as time-wise linearly varying parameters.

Indeed, $\gamma$ regulates the rate of growth of the recovered and has an ascending trend for all regions, while $\mu$ dictates the rate of change of the deceased which shows, conversely, a descending trend. Moreover, this model exhibits a piecewise constant trend for the contact rate $\beta$, in three distinct time windows. Notwithstanding the complexity of the parameter identification of this model, reasonable fittings were found for the daily positive cases, deceased, and recovered and definitely plausible predictions for the asymptomatic cases. The multi-scale regional epidemic analysis showed that Lombardia was characterized by the fastest growing epidemic spreading, and the most significant density of total positive cases with respect to its own population, followed by Emilia-Romagna, Piemonte, and Veneto, all neighboring and well-connected regions. Well spaced from these northern regions of Italy, we found the central regions of Marche, Toscana, and Lazio.

We corroborated our observations by making use of hierarchical clustering and multidimensional analyses which uncovered the similarities/dissimilarities between the different regional scales in comparison with the macropandemic scale. The different speeds of spreading between the more densely populated northern regions and the central regions, or even more evidently, the southern regions of Italy together with the strict national lockdown and closure of regional and transnational borders, were the key to slow down substantially or even prevent the spreading in the initially less affected regions of the country. We believe that a deeper and more sophisticated multi-scale analysis is possible, using the eigensolutions of the A-SIRD model for each region, to achieve the quantification of local regional-scale epidemic participation factors which suitably combine the meaningful set of epidemiological variables.
